# The ZIP8/SIRT1 axis regulates alveolar progenitor cell renewal in aging and idiopathic pulmonary fibrosis

**DOI:** 10.1172/JCI157338

**Published:** 2022-06-01

**Authors:** Jiurong Liang, Guanling Huang, Xue Liu, Forough Taghavifar, Ningshan Liu, Yizhou Wang, Nan Deng, Changfu Yao, Ting Xie, Vrishika Kulur, Kristy Dai, Ankita Burman, Simon C. Rowan, S. Samuel Weigt, John Belperio, Barry Stripp, William C. Parks, Dianhua Jiang, Paul W. Noble

**Affiliations:** 1 Department of Medicine and Women’s Guild Lung Institute and; 2 Genomics Core, Cedars-Sinai Medical Center, Los Angeles, California, USA.; 3 Department of Medicine, UCLA, Los Angeles, California, USA.; 4 Department of Biomedical Sciences, Cedars-Sinai Medical Center, Los Angeles, California, USA.

**Keywords:** Pulmonology, Stem cells, Adult stem cells, Fibrosis

## Abstract

Type 2 alveolar epithelial cells (AEC2s) function as progenitor cells in the lung. We have shown previously that failure of AEC2 regeneration results in progressive lung fibrosis in mice and is a cardinal feature of idiopathic pulmonary fibrosis (IPF). In this study, we identified deficiency of a specific zinc transporter, *SLC39A8* (ZIP8), in AEC2s from both IPF lungs and lungs of old mice. Loss of ZIP8 expression was associated with impaired renewal capacity of AEC2s and enhanced lung fibrosis. ZIP8 regulation of AEC2 progenitor function was dependent on SIRT1. Replenishment with exogenous zinc and SIRT1 activation promoted self-renewal and differentiation of AEC2s from lung tissues of IPF patients and old mice. Deletion of Zip8 in AEC2s in mice resulted in impaired AEC2 renewal, increased susceptibility to bleomycin injury, and development of spontaneous lung fibrosis. Therapeutic strategies to restore zinc metabolism and appropriate SIRT1 signaling could improve AEC2 progenitor function and mitigate ongoing fibrogenesis.

## Introduction

Idiopathic pulmonary fibrosis (IPF) is a fatal form of interstitial lung disease (ILD), with most patients dying or requiring a lung transplant within 3–5 years of diagnosis (1). The etiology is unknown, although several potential risk factors, such as aging, cigarette smoking, gastroesophageal reflux, as well as environmental factors, have been suggested (1). The hallmarks in the pathogenesis of IPF are repeated epithelial cell injury and inadequate alveolar epithelial repair. Injured epithelial cells activate fibroblasts through epithelial-mesenchymal crosstalk (2, 3), leading to excessive extracellular matrix production and lung fibrosis (4, 5). Inflammation and immunity also play a role in IPF pathogenesis (6–9).

Type 2 alveolar epithelial cells (AEC2s) function as progenitor cells that maintain epithelium homeostasis and repair damaged epithelium after injury (10–12). Repeated injury impairs the renewal capacity of AEC2s. We and others have previously shown that AEC2 levels were reduced in lungs of patients with IPF (13, 14). The remaining AEC2s in the IPF lung had impaired progenitor function (13). However, the molecular mechanisms that control AEC2 stem cell renewal and unremitting lung fibrosis remain poorly understood.

IPF is a disease of aging (15), although younger patients may be affected with familial pulmonary fibrosis (16). The incidence, prevalence, and mortality of IPF increase with aging (17, 18). Phenotypes of cellular aging, including epithelial cell apoptosis (19, 20), autophagy (21), senescence (22), telomere shortening (23, 24), mitochondria dysfunction (25, 26), and oxidative stress (27), are all observed in IPF lungs. Although the concept that IPF is a disease of aging has been widely accepted (15), there are limited studies focusing on the specific age-related mechanisms that contribute to the development and progression of IPF. In this study, we provide insights into the detrimental role of aging on AEC2 progenitor renewal in IPF and aged mouse lungs.

Multiple metabolic dysfunctions occur with aging (28). Recent studies have linked metabolic changes with stem cell fate (29). Zinc is an essential element and is involved in maintaining multiple metabolic functions (30). Reports have implicated zinc deficiency in exacerbating ventilator-induced lung injury (31, 32). Zinc deficiency increased lung and spleen damage in animal sepsis models (33). These data suggest the importance of zinc metabolism in lung biology, particularly in response to tissue injury. Zinc homeostasis is controlled by 2 zinc transporter families — Zn transporters (ZnT) and Zrt-, Irt-related proteins (ZIP) — in maintaining zinc mobilization and compartmentalization across biological membranes (34). Studies have shown that zinc and zinc transporters play an important role in maintenance of homeostasis and progenitor function of intestinal epithelium (35). Zinc is required for sirtuin signaling, which is a major cell energy metabolism pathway (28). Studies have shown that sirtuins play a role in stem cell maintenance and differentiation (36). The role of zinc metabolism and sirtuin signaling in lung stem cell renewal and epithelial repair is largely unknown.

In this study, using unbiased single-cell RNA sequencing (scRNA-Seq) with the goal of determining key signaling pathway defects in IPF AEC2s that fail to regenerate, we identified several dysregulated metabolic pathways. We discovered an unexpected significant decrease in the level of zinc transporter *SLC39A8* (ZIP8) in IPF AEC2s. We further demonstrated that loss of ZIP8 in IPF AEC2s resulted in impaired progenitor function that was dependent on the sirtuin SIRT1. Exogenous zinc treatment increased ZIP8 and SIRT1 expression and promoted the renewal capacity of AEC2s. Using a mouse model of aging, we found that AEC2s from bleomycin-injured old mouse lungs and IPF AEC2s had similar genomic changes and phenotypes. AEC2s from the lungs of old mice showed reduced Zip8 expression and decreased renewal capacity relative to AEC2s from the lungs of young mice. Exogenous zinc was able to promote renewal and differentiation of murine AEC2s, while the response to zinc treatment was blunted with aging. Using genetically modified mice, we further demonstrated that deletion of ZIP8 selectively in the adult AEC2 compartment impaired AEC2 recovery and resulted in spontaneous fibrosis in aged mice, and increased lung fibrosis after bleomycin injury. To our knowledge, this is the first evidence to show that ZIP8, zinc metabolism, and SIRT1 play a role in regulating AEC2 progenitor renewal and lung fibrosis.

## Results

### Decreased expression of a specific zinc transporter, ZIP8, in IPF AEC2s.

**Figure 1:**
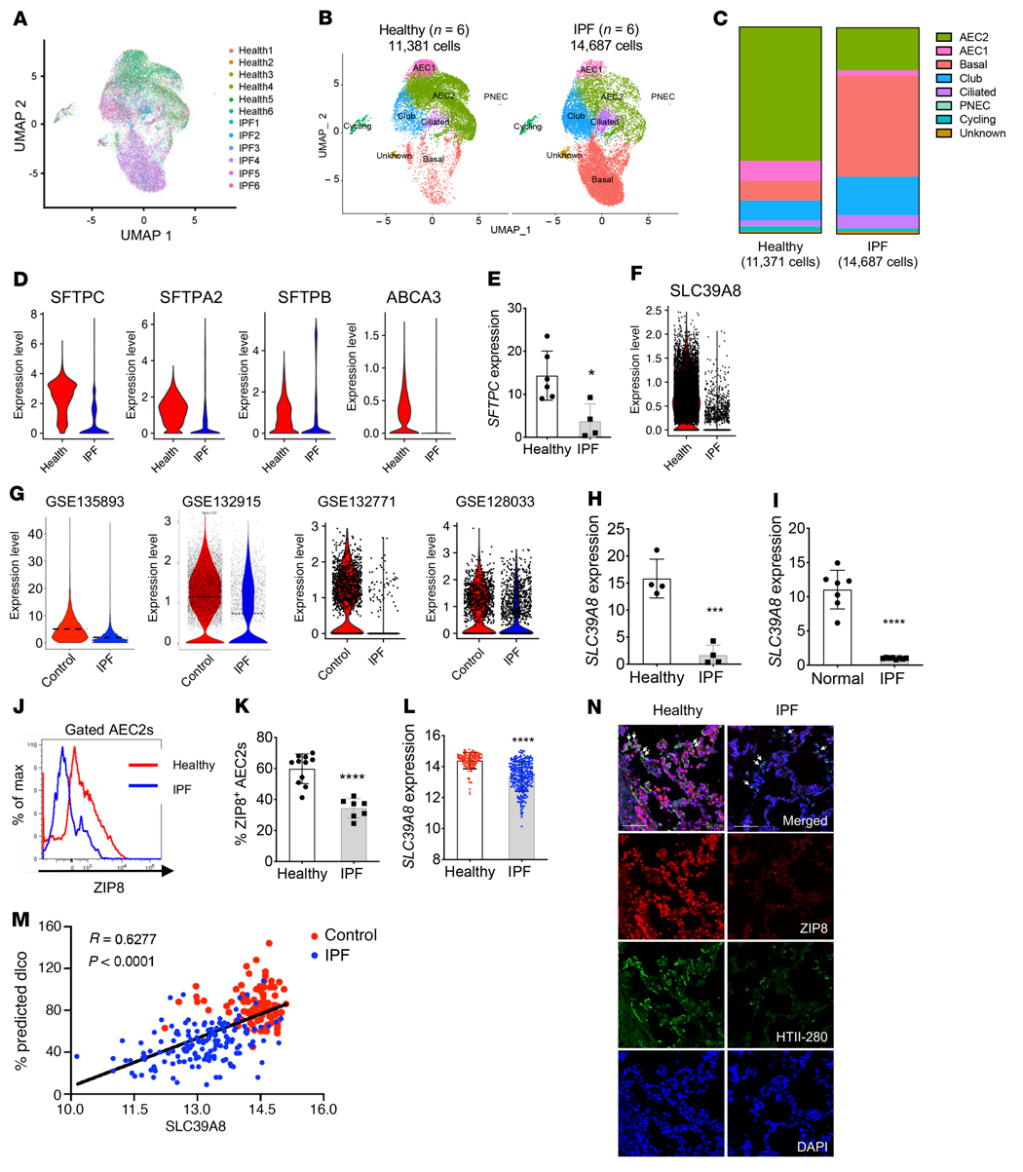
Selective loss of ZIP8 in IPF AEC2s. (**A**) UMAP plots of flow-enriched EpCAM^+^CD31^–^CD45^–^ cells from healthy (11,381 cells, *n* = 6) and IPF lungs (14,687 cells, *n* = 6). (**B** and **C**) Clusters of epithelial cell types (**B**) and distribution of epithelial cell types (**C**) in healthy and IPF lungs. PNEC, pulmonary neuroendocrine cells. (**D**) Expression of AEC2 marker genes in cells from healthy and IPF lungs. (**E**) *SFTPC* expression in healthy (*n* = 6) and IPF AEC2s (*n* = 4) by qPCR (*P < 0.05). (**F**) Expression of the zinc transporter gene *SLC39A8* in AEC2s from healthy and IPF lungs in the present scRNA data set. (**G**) Expression of *SLC39A8* in AEC2s from healthy (control) and IPF lungs from the recently published data sets GSE135893, GSE132915, GSE132771, and GSE128033. (**H** and **I**) qPCR for *SLC39A8* mRNA expression in AEC2s freshly isolated from lung tissues (*n* = 4 each, ***P < 0.001) (**H**) and derived from 3D-cultured organoids (healthy *n* = 7, IPF *n* = 8, ****P < 0.0001) (**I**). (**J** and **K**) Flow cytometry of cell-surface ZIP8 levels and percentage of ZIP8^+^ cells in healthy (*n* = 11) and IPF (*n* = 7) AEC2s (****P < 0.0001). (**L**) *SLC39A8* expression in healthy (*n* = 108) and IPF lung tissues (*n* = 160) (****P < 0.0001). Data are shown as mean ± SEM. (**M**) Correlation of *SLC39A8* expression and lung function as DLCO (% predicted DLCO) in healthy control (*n* = 97) and IPF (*n* = 145) lung tissues (*r* = 0.6277). (**N**) Immunofluorescence staining for the AEC2 marker HTII-280 and ZIP8. Arrows indicate examples of HTII-280^+^ cells. Scale bars, 100 μm. Staining was performed with lung sections from 3 IPF patients and 3 healthy donors. **E**, **H**, **I**, **K**, and **L**: unpaired 2-tailed Student’s *t* test; **M**: nonparametric Spearman’s correlation analysis.

In a previous study, we found a significant reduction in the AEC2 population in lungs of patients with IPF (13). Interestingly, the remaining AEC2s in the diseased lung had impaired renewal capacity compared with healthy AEC2s (13). To further investigate the genomic alterations that may be linked to AEC2 progenitor failure in IPF, we performed scRNA-Seq of freshly isolated flow cytometry–enriched epithelial cells (CD31^–^CD45^–^EpCAM^+^) from explanted lungs of patients with IPF and from healthy donors. We processed 11,381 cells from 6 healthy individuals and 14,687 cells from 6 IPF patients, after removing cells with low-quality RNA. Uniform Manifold Approximation and Projection (UMAP) plots were generated with a Seurat package (37, 38), and there was a high degree of overlap among all samples (Figure 1A). The major lung epithelial cell types were readily identified with classical cell markers (Figure 1B).

We observed far fewer cells in the AEC2 cluster among the IPF compared with the healthy donor cell samples (Figure 1, B and C). Increased levels of basal-like and ciliated cells were observed with the cells from IPF lungs (Figure 1, B and C), consistent with recent reports (14, 39, 40). Expression of AEC2 marker genes including *SFTPC*, *SFTPA2*, *SFTPB*, and *ABCA3* was downregulated in IPF AEC2s (Figure 1D). We confirmed the decreased *SFTPC* expression in flow-sorted AEC2s from IPF lungs relative to AEC2s from healthy donor lungs using real-time quantitative PCR (qPCR) (Figure 1E).

Next, we examined the genes differentially expressed in AEC2s from lungs of IPF patients and healthy donors. It was striking that multiple zinc metabolism–related genes, including *MT1E*, *MT2A*, *GCLM*, and *GSR*, were downregulated in IPF AEC2s (Supplemental Figure 1A; supplemental material available online with this article; https://doi.org/10.1172/JCI157338DS1). This finding led us to further investigate zinc metabolism in IPF AEC2s.

Zinc homeostasis is controlled by zinc transporters (34). Most zinc transporters are tissue and cell type specific (34). There are a total of 14 members of the ZIP protein family, which are zinc influx transporters encoded by *SLC39* family genes. Our scRNA-Seq data showed that among the *SLC39* family genes, *SLC39A8* (encoding ZIP8) was expressed at the highest level in healthy AEC2s (Supplemental Figure 1B). Most interestingly, expression of *SLC39A8* was dramatically decreased in IPF AEC2s, prompting further investigations (Figure 1F and Supplemental Figure 1B). We next analyzed recently published data sets from studies performed with scRNA-Seq on samples from patients with IPF. *SLC39A8* was significantly decreased in IPF AEC2s compared with healthy AEC2s in NCBI Gene Expression Omnibus data sets GSE135893 (40), GSE132915 (38), GSE132771 (41), and GSE128033 (42) (Figure 1G).

Next we analyzed data sets published by Fagerberg et al. (43) and confirmed that expression of *SLC39A8* was higher in human lungs than that of other *SLC39* family genes (Supplemental Figure 1C), and lung tissue expressed *SLC39A8* at higher levels than other organs and tissues (Supplemental Figure 1D). We extended our investigation to determine the cell types that express *SLC39A8* in the lung by analyzing our scRNA-Seq data for both human lung epithelial cells and fibroblasts (37). We found that *SLC39A8* was mainly expressed in EpCAM^+^ epithelial cells and its expression in EpCAM^–^ mesenchymal cells was very low (Supplemental Figure 1E). Within lung epithelial cells, *SLC39A8* was mainly expressed in AEC2s and AEC1s (Supplemental Figure 1F). *SLC39A8* expression was also decreased in AEC1s from IPF lungs relative to AEC1s from healthy lungs (Supplemental Figure 1G). The significance of the downregulation of *SLC39A8* in AEC1s from IPF lungs is under investigation and was not addressed in this study.

We confirmed decreased *SLC39A8* expression in IPF AEC2s from both freshly isolated (Figure 1H) and 3D-cultured organoids (Figure 1I) using qPCR. Flow cytometry showed that cell-surface ZIP8 expression of IPF AEC2s was much lower than that of healthy AEC2s (Figure 1, J and K). We further analyzed data deposited to the Lung Genomics Research Consortium (LGRC; ref. 44) and confirmed that *SLC39A8* expression was significantly lower in lung tissues of patients with IPF (Figure 1L) and was positively correlated with carbon monoxide diffusing capacity (DLCO; Figure 1M). Using immunofluorescence staining, we demonstrated that AEC2s in IPF lung sections had lower ZIP8 expression than AEC2s from healthy donors (Figure 1N and Supplemental Figure 2A).

To determine whether the decreased ZIP8 expression in IPF AEC2s was affected by the therapeutic drugs the patients took before transplantation, we treated freshly isolated single cells from healthy donor lungs with pirfenidone and nintedanib for 48 hours and then assessed ZIP8 expression levels on AEC2s using flow cytometry. Our results showed that neither drug had an effect on ZIP8 expression of AEC2s (Supplemental Figure 2, B and C).

The *SLC30* gene family encodes another group of zinc transporters, ZNT proteins. The function of ZNT proteins involves exporting zinc to the extracellular space or transferring it into intracellular compartments when cellular zinc levels rise (34). Expression of *SLC30* family genes was generally low in human AEC2s and not significantly different between healthy and IPF AEC2s (Supplemental Figure 1H).

### ZIP8-dependent zinc metabolism is required for AEC2 renewal.

**Figure 2:**
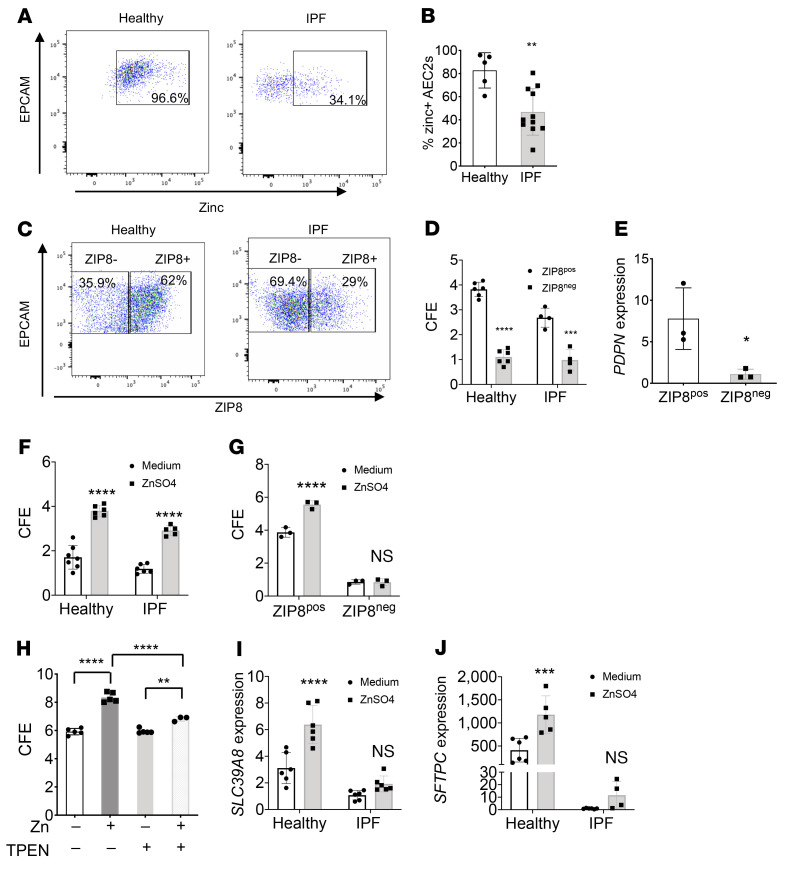
ZIP8-dependent zinc metabolism is required for AEC2 renewal. (**A**) Representative plots of intracellular zinc in gated healthy and IPF AEC2s by flow cytometry. (**B**) Percentage of zinc-positive AEC2s within the gated AEC2 population from healthy (*n* = 5) and IPF lungs (*n* = 11) (**P < 0.01). (**C**) Flow cytometry plots of gated ZIP8^+^ and ZIP8^–^ AEC2s. (**D**) CFE of flow-sorted ZIP8^+^ and ZIP8^–^ AEC2s from healthy (*n* = 6) and IPF lungs (*n* = 4) (***P < 0.001, ****P < 0.0001). (**E**) *PDPN* expression in AEC2s derived from 3D-cultured organoids (*n* = 3, *P < 0.05). (**F**) CFE of AEC2s from healthy and IPF lungs with and without ZnSO_4_ (100 μM) treatment (*n* = 5–7, ****P < 0.0001). (**G**) CFE of ZIP8^+^ and ZIP8^–^ AEC2s with and without ZnSO_4_ (100 μM) treatment (*n* = 3 each, ****P < 0.0001). (**H**) CFE of AEC2s with and without ZnSO_4_ and TPEN (1 μM) treatment (*n* = 3–5, **P < 0.01, ****P < 0.0001 by ANOVA). (**I** and **J**) Expression of *SLC39A8* (*n* = 4–6, ****P < 0.0001) and *SFTPC* (*n* = 4–6, ***P < 0.001) in AEC2s with and without ZnSO_4_ treatment by qPCR. Data are shown as mean ± SEM. **B** and **E**: unpaired 2-tailed Student’s *t* test; **D**, **F**, **G**, **H**, **I**, and **J**: 2-way ANOVA.

We anticipated that IPF AEC2s might have impaired zinc uptake capacity due to loss of the zinc influx transporter ZIP8. To test this hypothesis, we incubated single-cell lung homogenates from IPF patients and healthy donors in the presence of 100 μM zinc sulfate (ZnSO_4_) overnight and then stained the cells for AEC2 markers and intracellular zinc for flow cytometry. Our data showed that IPF AEC2s had much lower intracellular zinc levels than AEC2s from healthy lungs (Figure 2, A and B, and Supplemental Figure 3). These findings represent the first evidence to our knowledge of zinc metabolic dysregulation in IPF AEC2s characterized by the loss of a zinc transporter, *SLC39A8*/ZIP8, and impaired zinc uptake.

Zinc is an essential element for cell metabolism and it is involved in a variety of cell functions, including maintaining epithelial progenitor function (35, 45, 46). We hypothesized that ZIP8, as the main zinc influx transporter of AEC2s, would have a role in regulating AEC2 progenitor function. To test this hypothesis, we sorted ZIP8^+^ and ZIP8^–^ AEC2s from both healthy and IPF lungs by flow cytometry (Figure 2C) and examined the renewal capacity of the progenitor cells as measured by colony-forming efficiency (CFE; ref. 47) with 3D organoid cultures. ZIP8^–^ AEC2s showed significantly reduced CFE compared with ZIP8^+^ AEC2s from the same lung (Figure 2D). Cells from both healthy and IPF lungs contained ZIP8^+^ and ZIP8^–^ populations, and we found a much lower percentage of ZIP8^+^ AEC2s in IPF lungs (Figure 2C). Interestingly, ZIP8^+^ AEC2s from IPF lungs had lower CFE than ZIP8^+^ AEC2s from healthy lungs (Figure 2D), indicating there are likely other factors contributing to impaired renewal of IPF AEC2s. ZIP8^–^ AEC2s derived from 3D-cultured organoids showed decreased *PDPN* expression (Figure 2E), suggesting impaired differentiation of the cells with ZIP8 deficiency.

Next we tested whether exogenous zinc impacts AEC2 progenitor renewal. In a proof-of-principle experiment, we applied 100 μM ZnSO_4_ to 3D organoid cultures of AEC2s; zinc treatment improved the colony-forming capacity of AEC2s from both healthy and IPF lungs (Figure 2F). However, zinc treatment showed no effect on CFE of ZIP8^–^ AEC2s (Figure 2G), indicating that ZIP8 might act as the sole functional zinc transporter of AEC2s. To verify the specificity of exogenous zinc treatment on promoting AEC2 progenitor renewal, we applied a zinc chelator, TPEN, to the 3D organoid culture. TPEN abolished zinc’s effect of promoting AEC2 renewal (Figure 2H).

Zinc treatment significantly elevated *SLC39A8* (Figure 2I) and *SFTPC* (Figure 2J) expression in AEC2s from healthy lungs. The same trend of effect was observed in AEC2s from IPF lungs (Figure 2, I and J), but the difference was not significant.

### Downregulation of sirtuin signaling in IPF AEC2s.

**Figure 3:**
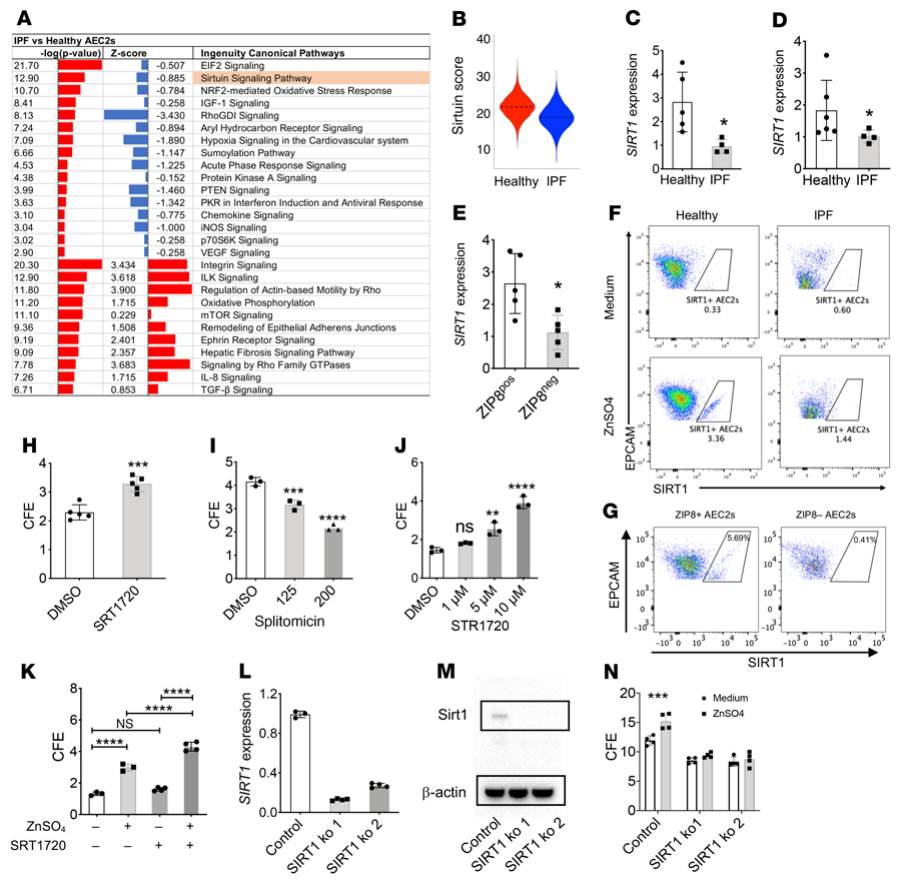
ZIP8-dependent zinc metabolism regulates AEC2 renewal through SIRT1. (**A**) IPA pathway analysis of AEC2s from healthy and IPF lungs.(**B**) Sirtuin activation score of healthy and IPF AEC2s. (**C** and **D**) *SIRT1* expression in freshly isolated AEC2s (**C**) and AEC2s derived from 3D organoids (**D**) by qPCR (*n* = 4–6, *P < 0.05). (**E**) *SIRT1* expression of AEC2s derived from 3D-cultured organoids of ZIP8^+^ and ZIP8^–^ AEC2s (*n* = 5 each, *P < 0.05). (**F**) Intracellular SIRT1 levels in healthy and IPF AEC2s without and with ZnSO_4_ treatment by flow cytometry (*n* = 3–4). (**G**) Intracellular SIRT1 levels in gated ZIP8^+^ and ZIP8^–^ AEC2s from healthy lungs (*n* = 4). (**H** and **I**) CFE with 3D organoid culture of AEC2s from healthy lungs treated with either 1 μM SRT1720 (*n* = 4–5, ***P < 0.001 by ANOVA) (**H**) or 125 and 200 μM splitomicin (*n* = 3, ***P < 0.001, ****P < 0.0001 by ANOVA) (**I**). (**J**) CFE of AEC2s from IPF lungs cultured with SRT1720 at the indicated concentrations (*n* = 3, **P < 0.01, ****P < 0.0001 by ANOVA). (**K**) CFE of AEC2s from IPF lungs cultured with 1 μM SRT1720, 100 μM ZnSO_4_, or both (*n* = 3–4, ****P < 0.0001 by ANOVA). (**L**–**N**) A549 cells with SIRT1 knockout and control cells. SIRT1 ko 1, set 1 sgRNA; SIRT ko 2, set 2 sgRNA. (**L**) *SIRT1* expression by qPCR. (**M**) SIRT1 expression by Western blot analysis; the same experiments were performed 3 times. (**N**) CFE with 3D organoid culture (*n* = 4, ***P < 0.001 by ANOVA). Data are shown as mean ± SEM. Unpaired 2-tailed Student’s *t* test.

In order to determine the pathways downstream of ZIP8 that regulate AEC2 progenitor renewal, we performed pathway analysis with the scRNA-Seq data and found that the sirtuin signaling pathway was among the most downregulated pathways in IPF AEC2s (Figure 3A). IPF AEC2s showed a lower SIRT activation score (Figure 3B). SIRT1 is a critical member of the sirtuin family (48, 49). By qPCR , we found that SIRT1 expression was decreased in both freshly isolated IPF AEC2s (Figure 3C) and IPF AEC2s derived from 3D-cultured organoids (Figure 3D).

### Zinc induces SIRT1 expression in human AEC2s in a ZIP8-dependent manner.

Sirtuin signaling is a zinc-dependent cell energy metabolic pathway (50), and SIRT enzymatic activities require an adequate level of intracellular zinc (36). We hypothesized that downregulated SIRT1 expression in IPF AEC2s might be associated with loss of ZIP8 and deficiency in intracellular zinc. To test this hypothesis, we isolated RNA from flow-sorted ZIP8^+^ and ZIP8^–^ AEC2s from healthy human lungs and compared SIRT1 expression. Our results showed that ZIP8^–^ AEC2s had much lower SIRT1 expression than ZIP8^+^ AEC2s (Figure 3E). To further demonstrate the correlation between ZIP8 and SIRT1 expression in AEC2s, we cultured single cells from both healthy and IPF human lungs with and without 100 μM ZnSO_4_ and measured SIRT1 expression in AEC2s by flow cytometry. SIRT1 expression in gated AEC2s was very low at baseline in both healthy and IPF AEC2s (Figure 3F). We observed a distinct SIRT1^+^ population within gated AEC2s from healthy lungs with zinc treatment. However, there were much fewer SIRT1^+^ ACE2s from IPF lungs, even though there was some increase with zinc treatment (Figure 3F). We then separated AEC2s from healthy lungs according to whether or not they expressed ZIP8 and found that the SIRT1^+^ population was mainly expressed in ZIP8^+^ AEC2s (Figure 3G). These results suggest that zinc-induced SIRT1 expression in AEC2s is ZIP8 dependent and that downregulation of SIRT1 in IPF AEC2s is due to ZIP8 deficiency.

### ZIP8 regulates AEC2 renewal through SIRT1.

The function of the sirtuin signaling pathway has been linked to stem cell renewal (36, 50, 51). To test whether sirtuin signaling plays a direct role in regulating AEC2 progenitor renewal, we applied a SIRT1 activator, SRT1720, and a SIRT1 inhibitor, splitomicin (splitomycin), to 3D organoid cultures of AEC2s from healthy lungs. Our results showed that SRT1720 promoted (Figure 3H) and splitomicin inhibited (Figure 3I) the progenitor renewal capacity of healthy human AEC2s. These data suggested that SIRT1 activation is directly associated with the progenitor function of AEC2s and that decreased SIRT1 expression in IPF AEC2s contributes to the impaired renewal capacity of the cells. Moreover, SIRT1 activation by SRT1720 improved the renewal capacity of IPF AEC2s (Figure 3J). We further observed an additive effect in promoting renewal of AEC2s from IPF lungs with the combination of ZnSO_4_ and the SIRT1 activator by 3D organoid culture (Figure 3K).

To further demonstrate that ZIP8 regulates AEC2 renewal through SIRT1, we used CRISPR/Cas9 to knock out SIRT1 in A549 cells. We confirmed successful knockout of SIRT1 by qPCR (Figure 3L) and Western blot analysis (Figure 3M; see the complete unedited gels in the supplemental material). Next, we flow sorted the subpopulation double positive for ZIP8 and HTII-280 (52) from SIRT1-KO and control A549 cells (Supplemental Figure 4A) and applied the cells to 3D organoid culture with and without zinc treatment. Zinc treatment increased CFE of control cells but not cells with SIRT1 deficiency (Figure 3N and Supplemental Figure 4B).

### Reduced ZIP8 expression and renewal capacity of AEC2s from old mouse lungs.

**Figure 4:**
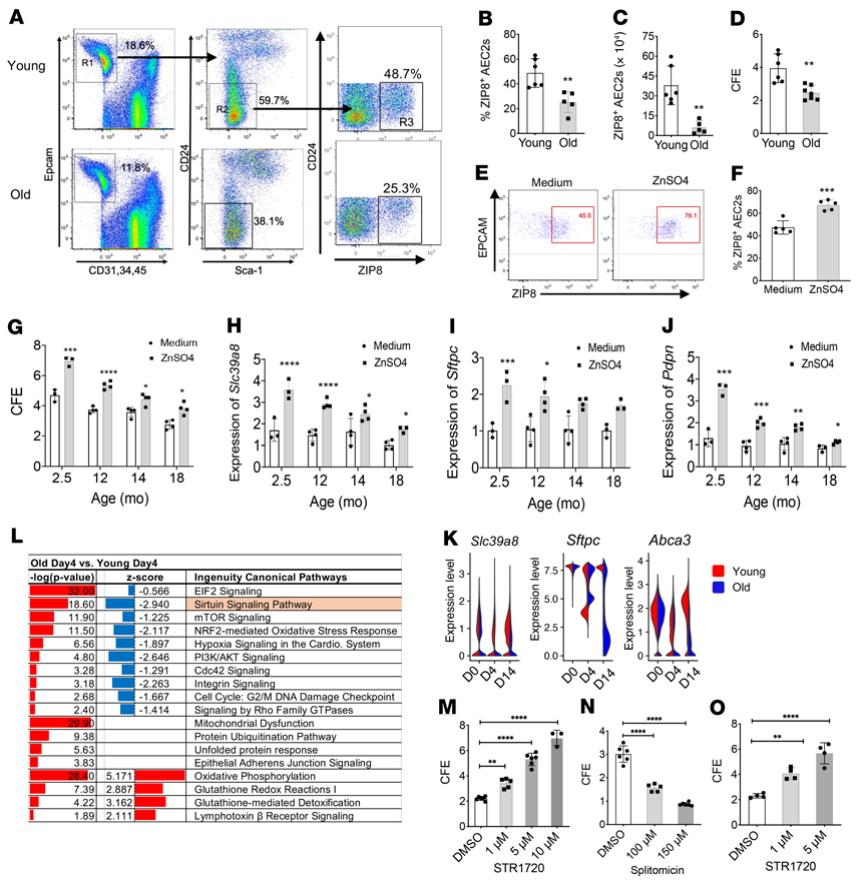
Downregulated ZIP8/SIRT1 signaling and decreased renewal capacity of AEC2s from old mouse lungs. (**A**) Flow cytometry analysis to gate out total AEC2s (R2), and ZIP8 expression AEC2s (R3) from lung homologies of young and old mice. (**B**) Percentage of ZIP8^+^ cells (R3) within total AEC2s (*n* = 5–6, **P < 0.01). (**C**) Number of ZIP8^+^ cells recovered from young and old mouse lung (*n* = 5–6, **P < 0.01). (**D**) CFE of mouse AEC2s isolated from young and old mouse lungs (*n* = 6–7, **P < 0.01). (**E** and **F**) Flow cytometry analysis of ZIP8 expression in gated AEC2s cultured with medium only or medium containing 100 μM ZnSO_4_ (*n* = 6, ***P < 0.001). (**G**–**J**) 3D organoid culture of AEC2s isolated from lungs of 2.5-, 12-, 14-, and 18-month-old mice with and without 100 μM ZnSO_4_ treatment. (**G**) CFE (*n* = 3–4, *P < 0.05, ***P < 0.001, ****P < 0.0001 by ANOVA). (**H**–**J**) Expression of *Slc39a8* (**H**), *Sftpc* (**I**), and *Pdpn* (**J**) in AEC2s derived from 3D-cultured organoids with and without ZnSO_4_ treatment by qPCR (*n* = 3–4, *P < 0.05, **P < 0.01, ***P < 0.001, ****P < 0.0001 by ANOVA). (**K**) Violin plots of gene expression in AEC2s from lungs of bleomycin-treated young and old mice. (**L**) IPA pathway analysis of AEC2s from young and old mice on day 4 after bleomycin injury. (**M** and **N**) CFE of AEC2s from uninjured 10- to 12-week-old young mice treated with SRT1720 (*n* = 3–6, **P < 0.01, ****P < 0.0001 by ANOVA) (**M**) and splitomicin (*n* = 5–6, ****P < 0.0001 by ANOVA) (**N**) at the indicated doses and DMSO control. (**O**) CFE of AEC2s from uninjured 20- to 24-month-old mice treated with SRT1720 at the indicated doses and DMSO control (*n* = 4, **P < 0.01, ****P < 0.0001 by ANOVA). Data are shown as mean ± SEM. Unpaired 2-tailed Student’s *t* test.

Aging is one of the important risk factors for IPF. We therefore examined the impact of aging on AEC2 progenitor function in mice. We assessed epithelial cells from the lungs of mice aged 2–3 months (young) and 18–20 months (old) by flow cytometry using a cell-surface marker gating strategy established in our laboratory (13). Epithelial cells were gated as CD31^–^CD34^–^CD45^–^EpCAM^+^ (R1 in Figure 4A), whereas AEC2s were gated as CD31^–^CD34^–^CD45^–^EpCAM^+^Sca-1^–^CD24^–^ (R2 in Figure 4A). The numbers of total cells recovered from each lung were similar in young and old mice (Supplemental Figure 5A). However, we observed markedly reduced percentages and total numbers of epithelial cells in old compared young mouse lungs (Supplemental Figure 5, B and C). We then examined AEC2s within the entire epithelial population and found that the percentage of AEC2s was severely reduced in lungs of old relative to young mice (Supplemental Figure 5D), resulting in extremely low numbers of AEC2s in old mouse lungs under homeostatic conditions (Supplemental Figure 5E).

We next analyzed ZIP8 expression in mouse AEC2s and observed a marked reduction in both the percentage (Figure 4, A and B) and number (Figure 4C) of ZIP8^+^ AEC2s in old mouse lungs. These data suggested that loss of ZIP8^+^ AEC2 progenitor cells might be one of the characteristics of lung aging. We confirmed decreased *Slc39a8* expression in AEC2s from old mouse lungs by qPCR (Supplemental Figure 5F).

We then compared the regenerative capacity of AEC2s from young and old mice by 3D organoid culture. AEC2s from old mice gave rise to fewer colonies than AEC2s from young mice (Figure 4D). AEC2s derived from 3D-cultured organoids of old mouse AEC2s showed lower expression of *Sftpc* (Supplemental Figure 5G) and *Pdpn* (Supplemental Figure 5H), indicating impaired renewal and differentiation of AEC2s with aging. Reduced number and lower renewal capacity of AEC2s from aged mouse lungs were reported previously (53). Here we further demonstrate that the decreased renewal capacity of old AEC2s might be due to loss of ZIP8.

### Blunted response to zinc treatment of AEC2s from old mouse lungs.

We then sought to determine whether exogenous zinc could affect ZIP8 expression by murine AEC2s. We applied ZnSO_4_ treatment to single-cell homogenates of uninjured young mouse lungs and measured cell-surface ZIP8 levels of AEC2s by flow cytometry. Zinc treatment elevated ZIP8 expression of AEC2s (Figure 4, E and F).

Next we investigated the effect of aging on the response of AEC2s to exogenous zinc treatment. We isolated AEC2s from mice of multiple age groups — 2.5 (young), 12, 14, and 18 (old) months — and applied the flow-sorted AEC2s to 3D organoid cultures with and without zinc treatment (Figure 4, G–J). Zinc treatment increased the CFE of AEC2s for all age groups (Figure 4G). However, young ACE2s had the strongest response to zinc treatment, and the responses diminished with age (Figure 4G). Zinc treatment also increased expression of *Slc39a8*, *Sftpc*, and *Pdpn* (Figure 4, H–J), indicating improved progenitor renewal and differentiation of the cells. The effects of zinc treatment on expression of these genes were diminished with age (Figure 4, H–J).

### Impaired AEC2 recovery in old mouse lungs after bleomycin injury.

We anticipated that decreased AEC2 progenitor renewal capacity in old mice would impair AEC2 recovery after lung injury. We performed scRNA-Seq of flow-enriched lung epithelial cells from both young and old mice at baseline (uninjured, day 0) as well as days 4 and 14 after bleomycin injury (54). We examined the dynamic change in *Slc39a8* expression in AEC2s following bleomycin injury in both young and old mice. *Slc39a8* expression was similar in AEC2s from young and old mice at baseline (Figure 4K), even though we observed decreased cell-surface ZIP8 levels with old AEC2s (Figure 4A). On day 4, the time point of maximal AEC2 injury after bleomycin treatment, *Slc39a8* expression was decreased in AEC2s from both young and old mice (Figure 4K). However, AEC2s from old mouse lungs had more severe loss of *Slc39a8* expression than did young AEC2s (Figure 4K). It is interesting that on day 14, during the epithelial recovery stage after bleomycin injury, *Slc39a8* expression in ACE2s from young mice was partially recovered, but expression in AEC2s of old mice remained low (Figure 4K). Consistent with the pattern of *Slc39a8* expression, AEC2 marker genes including *Sftpc* and *Abca3*, were highly expressed in both young and old AEC2s from day 0 intact lungs (Figure 4K), and these genes were downregulated in both young and old AEC2s on day 4 after bleomycin injury (Figure 4K). It is interesting that on day 14, expression of AEC2 marker genes in AEC2s from young mice was recovered, but their expression remained low in AEC2s from old mice (Figure 4K). These data suggest that severe loss of ZIP8 in AEC2s significantly hampers the recovery of AEC2 integrity in old mice after lung injury.

### Downregulation of sirtuin signaling in AEC2s from bleomycin-injured old mouse lungs.

Next we performed pathway analyses of scRNA-Seq data of AEC2s from young and old mouse lungs harvested on day 4 after bleomycin injury, which is the time point of active epithelial injury. We found that the sirtuin signaling pathway was the highly downregulated pathway with the lowest *z* score in AEC2s from old mice (Figure 4L). This finding is strikingly similar to what we have observed with IPF AEC2s, and it suggests that AEC2s in bleomycin-injured old mouse lungs might have gene signatures as well as progenitor function defects similar to those of IPF AEC2s. Similar to what we observed with human AEC2s, the sirtuin activator SRT1720 promoted (Figure 4M) and the sirtuin inhibitor splitomicin inhibited (Figure 4N) the renewal capacity of AEC2s from young mouse lungs in a dose-dependent manner. Next we tested the effect of SRT1720 on old AEC2s and found that SIRT1 activation by SRT1720 treatment increased colony formation of AEC2s from 20- to 24-month-old mice (Figure 4O).

Sirtuin signaling requires both zinc and NAD^+^. Studies have shown that NAD^+^ precursors improve stem cell function and prolong life span (28, 55). We extended our study to explore whether NAD^+^ metabolism is also dysregulated in IPF lungs. We found that several genes encoding NAD^+^ synthesis enzymes, including *NNMT*, *NAMPT*, *KYNU*, and *NQO1*, were downregulated in AEC2s from IPF relative to healthy lungs (Supplemental Figure 6A). Interestingly, the NAD^+^ precursors β-nicotinamide mononucleotide (NMN) and nicotinamide riboside (NR) increased colony formation of AEC2s from mouse lungs in 3D organoid culture (Supplemental Figure 6B).

### AEC2s with ZIP8 deletion showed impaired renewal and differentiation capacity and premature aging.

**Figure 5:**
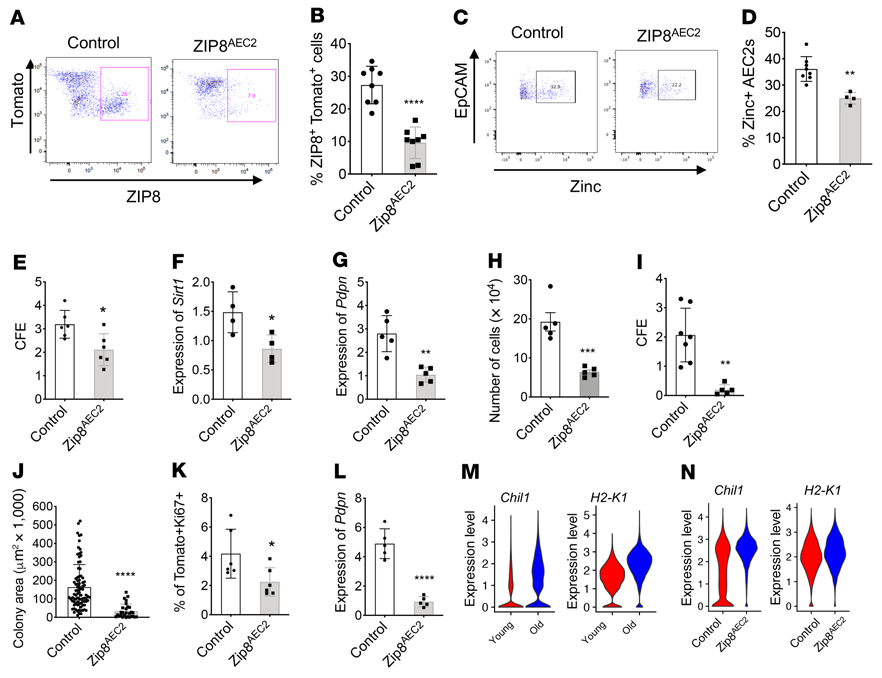
Targeted deletion of *Slc39a8* decreased AEC2 renewal. (**A**) ZIP-expressing cells among gated AEC2s and (**B**) percentage of ZIP8^+^ cells within the total AEC2 population from uninjured Zip8^AEC2^ and control mice by flow cytometry (*n* = 8, ****P < 0.001). (**C** and **D**) Intracellular zinc levels of AEC2s (**C**) and percentage of zinc^+^ AEC2s within the total AEC2 population (**D**) from Zip8^AEC2^ (*n* = 4) and control mice (*n* = 8) by flow cytometry (**P < 0.01). (**E**) CFE of flow-sorted AEC2s from uninjured Zip8^AEC2^ and control mice with 3D organoid culture (*n* = 6 each, *P < 0.05). (**F** and **G**) Expression of *Sirt1* (*n* = 4, *P < 0.05) (**F**) and *Pdpn* (*n* = 5, **P < 0.01) (**G**) in AEC2s derived from 3D-cultured organoids. (**H**–**J**) AEC2s from day 4 bleomycin-injured Zip8^AEC2^ and control mice. (**H**) Number of AEC2s recovered per lung (*n* = 5, ***P < 0.001). (**I** and **J**) CFE of AEC2s with 3D organoid culture (*n* = 5–7, **P < 0.01) (**I**) and colony size (*n* = 28–86, ****P < 0.0001) (**J**). (**K** and **L**) Ki-67 expression by flow cytometry (*n* = 6 each, *P < 0.05) (**K**) and *Pdpn* expression by qPCR (*n* = 5 each, ****P < 0.0001) (**L**) in AEC2s derived from 3D-cultured organoids. (**M** and **N**) Violin plots of gene expression in AEC2s with scRNA-Seq. (**M**) AEC2s from 2-month-old (Young) and 18- to 20-month-old (Old) C57BL/6 WT mice (*n* = 3). (**N**) AEC2s from 10- to 12-week-old (young) Zip8^AEC2^ mice and littermate controls 2 weeks after 4 doses of tamoxifen injection. Data are shown as mean ± SEM. Unpaired 2-tailed Student’s *t* test.

To illustrate the role of ZIP8 in AEC2 renewal and lung fibrosis in vivo, we generated a mouse line with targeted deletion of *Slc39a8* in the adult AEC2 compartment — *SFTPC-CreER Rosa26-tdTomato Zip8^fl/fl^* (Zip8^AEC2^) — by crossbreeding *Slc39a8*-floxed mice with *SFTPC-CreER Rosa26-tdTomato* mice. Rosa26-tdTomato is used to facilitate cell sorting. Upon tamoxifen treatment, *Slc39a8* expression in *Sftpc*-expressing cells is deleted. We confirmed the genotype of the of Zip8^AEC2^ mice with PCR (Supplemental Figure 7A). The Zip8^AEC2^ mice developed normally, without obvious physical defects. Deletion of ZIP8 did not cause changes in gene expression of other ZIP proteins, such as ZIP7, in AEC2s from Zip8^AEC2^ mice and littermate controls (Supplemental Figure 7B). We examined the lungs of 16-week-old young Zip8^AEC2^ mice and littermate controls 2 weeks after 4 doses of tamoxifen injection. Zip8^AEC2^ mice showed lung histology similar to that of littermate controls, without obvious lung inflammation and fibrosis (Supplemental Figure 7C). Total numbers of AEC2s recovered from lungs were not significantly different between Zip8^AEC2^ mice and littermate controls (Supplemental Figure 7D). However, levels of ZIP8^+^ cells within Tomato^+^ AEC2s were significantly reduced in the lungs of Zip8^AEC2^ mice (Figure 5, A and B), confirming successful deletion of ZIP8 in AEC2s. AEC2s from Zip8^AEC2^ mice showed decreased intracellular zinc levels after overnight culture in medium containing 100 μM ZnSO_4_ (Figure 5, C and D), indicating impaired zinc uptake of the cells with ZIP8 deletion.

We then examined the progenitor function of AEC2s with ZIP8 deletion. Our results showed that AEC2s isolated from uninjured Zip8^AEC2^ mice relative to littermate controls had reduced CFE with 3D organoid culture (Figure 5E). Expression of *Sirt1* (Figure 5F) and *Pdpn* (Figure 5G) in AEC2s derived from organoids with ZIP8 deletion was decreased. These data suggested that short-term deletion of AEC2 ZIP8 in young mice impaired progenitor function of AEC2s without causing immediate lung inflammation and fibrosis.

Next we treated young Zip8^AEC2^ mice and littermate controls with 2.5 U/kg bleomycin following 4 doses of tamoxifen injection and sacrificed the mice on day 4 after bleomycin treatment to examine AEC2s in the lungs. The number of AEC2s recovered from lungs of bleomycin-injured Zip8^AEC2^ mice was much smaller than that from littermate controls (Figure 5H). AEC2s from bleomycin-injured Zip8^AEC2^ mouse lungs showed severe regenerative defects with 3D organoid culture, and they gave rise to far fewer and smaller colonies than did AEC2s from littermate controls (Figure 5, I and J). AEC2s derived from 3D organoids with ZIP8 deletion showed reduced percentages of Tomato^+^Ki-67^+^ cells (Figure 5K) and a dramatic decrease in *Pdpn* expression (Figure 5L).

The phenotypes of AEC2s from 10- to 12-week-old Zip8^AEC2^ mice were strikingly like those of AEC2s from 18- to 20-month-old C57BL/6 WT mice. Next, we asked whether ZIP8 deletion causes accelerated AEC2 aging. We examined the expression of aging-related genes (56, 57) in AEC2s in our scRNA-Seq data sets generated with lung epithelial cells from young and old C57BL/6 WT mice (54) and from young Zip8^AEC2^ and control mice. We found that the aging-related genes — including the chitinase gene *Chil1* and the class I histocompatibility complex gene *H2-K1* — that were upregulated in AEC2s from 18- to 20-month-old WT mice (Figure 5M) were also upregulated in AEC2s from 12-week-old Zip8^AEC2^ mice 2 weeks after 4 doses of tamoxifen injection (Figure 5N). These data suggest that ZIP8 deficiency in AEC2s may lead to accelerated aging of the cells.

### Aged Zip8^AEC2^ mice developed spontaneous lung fibrosis.

**Figure 6:**
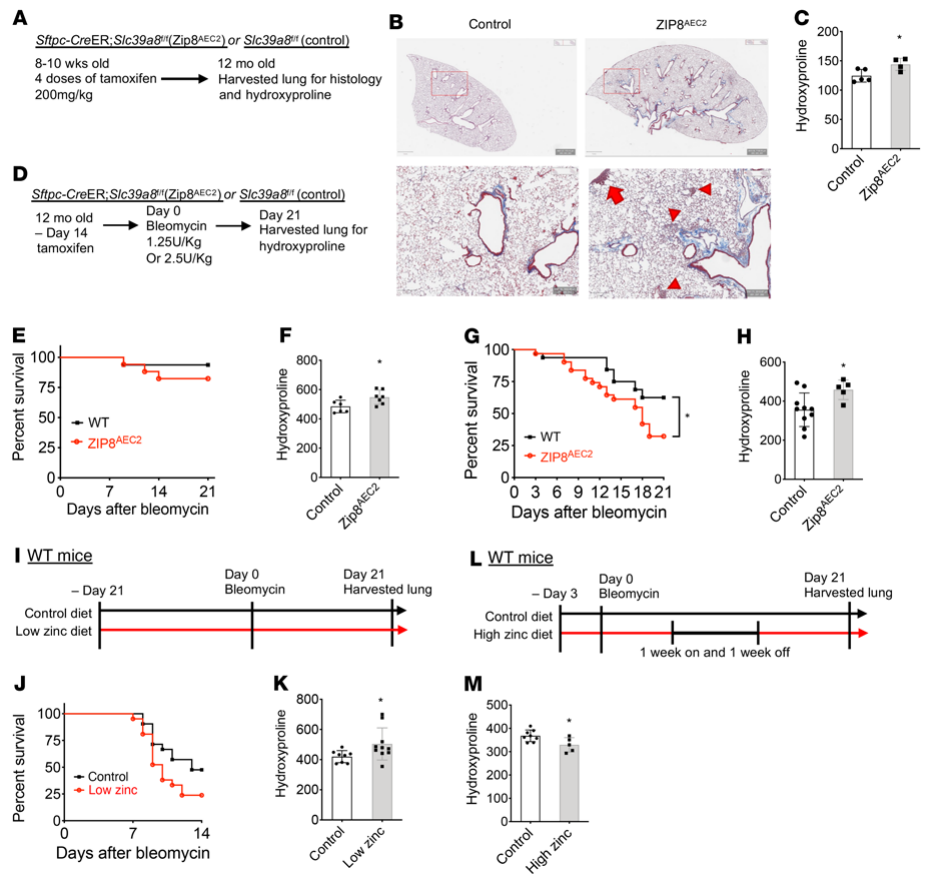
Spontaneous and increased lung fibrosis after bleomycin injury in old Zip8^AEC2^ mice and zinc metabolism–regulated lung fibrosis. (**A**) Experimental layout for spontaneous lung fibrosis in 12-month-old Zip8^AEC2^ and control mice. (**B**) Trichrome staining of lung sections showed that Zip8^AEC2^ mice developed fibrosis in subpleural (arrow) and interstitial (arrowheads) regions. Scale bars: top: 1 mm; bottom: 200 μm. (**C**) Hydroxyproline content (μg per right lung) in the lungs of 12-month-old male Zip8^AEC2^ (*n* = 4) and control (*n* = 5) mice (*P < 0.05). (**D**) Experimental layout for Zip8^AEC2^ and control mice treated with bleomycin following tamoxifen injection. (**E** and **F**) Survival (**E**) and hydroxyproline levels (μg per whole lung) (**F**) of 12-month-old Zip8^AEC2^ and control mice 21 days after 1.25 U/kg bleomycin treatment (**E**: *n* = 16–18, *P* = 0.33; **F**: *n* = 8–10, *P < 0.05). (**G** and **H**) Survival (**G**)and hydroxyproline content (μg per right lung) (**H**) of 7- to 10-month-old Zip8^AEC2^ and control mice on day 21 after 2 U/kg bleomycin treatment (**G**: *n* = 32, *P < 0.05; **H**: *n* = 5–10, *P < 0.05). (**I**) Experimental layout for WT mice fed low-zinc and control diets and treated with bleomycin for lung fibrosis study. (**J**) Survival of mice fed low-zinc and control diets on day 14 after bleomycin injury (*n* = 16–18, *P* = 0.07). (**K**) Hydroxyproline content (μg per right lung) of lungs from mice fed low-zinc and control diets on day 21 after bleomycin injury (*n* = 8–10, *P < 0.05). (**L**) Experimental layout for WT mice treated with high-zinc and control diets and bleomycin for lung fibrosis study. (**M**) Hydroxyproline content (μg per right lung) of lungs from mice fed high-zinc and control diets on day 21 after bleomycin injury (*n* = 5–8, *P < 0.05). Data are shown as mean ± SEM. **C**, **F**, **H**, **K**, and **M**: unpaired 2-tailed Student’s *t* test; **E**, **G**, and **J**: log-rank test.

Next we accessed the long-term effects of ZIP8 deletion in AEC2s on lung fibrosis. We injected 4 doses of 200 mg/kg tamoxifen into 8- to 10-week-old Zip8^AEC2^ and littermate controls and examined lung fibrosis when the mice were 12 months old (Figure 6A). The 12-month-old Zip8^AEC2^ mice developed spontaneous lung fibrosis, with increased trichrome staining (Figure 6B) and hydroxyproline content (Figure 6C). Lung fibrosis was observed in the subpleural and interstitial regions (Figure 6B).

### Increased susceptibility of Zip8^AEC2^ mice to bleomycin lung injury.

We next investigated lung fibrosis in Zip8^AEC2^ mice with bleomycin lung injury. To better represent the nature of IPF disease, which more commonly occurs in elderly male patients, we used old male mice for the experiments. First, we treated 10-month-old mice with low-dose (1.25 U/kg) bleomycin 2 weeks after tamoxifen injection and harvested the lungs 21 days after bleomycin injury (Figure 6D). Most Zip8^AEC2^ and control mice survived to the end of the experiment (Figure 6E). However, Zip8^AEC2^ mice showed increased hydroxyproline content compared with control mice (Figure 6F). Next, we treated the mice with higher-dose, 2 U/kg bleomycin, and Zip8^AEC2^ mice showed shortened survival (Figure 6G) and increased hydroxyproline content (Figure 6H). These data demonstrate that ZIP8 deletion in AEC2s increased susceptibility of the mice to bleomycin injury and resulted in worsened lung fibrosis.

### Zinc regulates lung fibrosis.

To further confirm that zinc deficiency worsens lung fibrosis, we fed WT mice a zinc-deficient diet (20 parts per million [ppm] zinc; refs. 32, 33, 58) versus a control diet (80 ppm zinc) for 3 weeks before bleomycin injury (2.5 U/kg), and mice were kept on the same diet until day 21 after bleomycin treatment(Figure 6I). Mice fed a zinc-deficient diet showed reduced survival (Figure 6J) and developed worse lung fibrosis and increased hydroxyproline contents compared with the mice on a control diet (Figure 6K). In additional experiments, we fed mice a diet containing higher levels of zinc (120 ppm) for 3 days before and then 1 week on and 1 week off after bleomycin injury. Control mice received a control diet with 80 ppm zinc (Figure 6L). Mice fed the higher-zinc diet showed a decrease in lung fibrosis (Figure 6M). These findings further support a role for zinc metabolism in regulating lung fibrosis.

## Discussion

**Figure 7:**
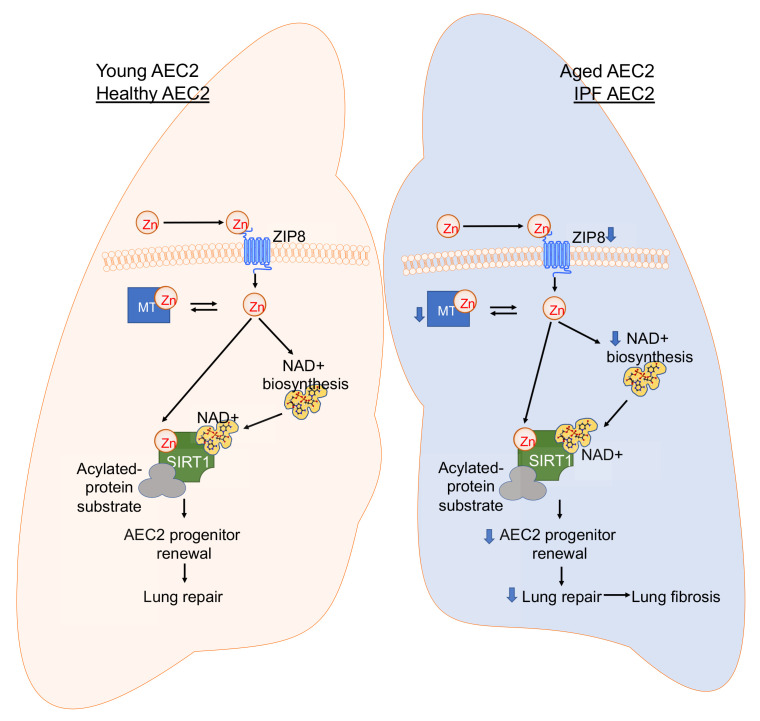
Summary of the role of the ZIP8/SIRT1 axis in regulating alveolar progenitor cell renewal. In young and healthy AEC2s, sufficient ZIP8 ensures adequate levels of intracellular zinc, SIRT1 activity, and AEC2 renewal capacity. However, in old AEC2s and IPF AEC2s, severely downregulated ZIP8 results in intracellular zinc deficiency and defective SIRT1 activity, which impairs AEC2 renewal. In addition, enzymes regulating NAD^+^ synthesis were downregulated in IPF AEC2s, further exaggerating SIRT1 impairment. Therefore, the optimal combinations of zinc, NAD^+^, and SIRT1 activation may restore AEC2 integrity and mitigate fibrosis. MT, metallothionein.

The molecular mechanisms that regulate AEC2 progenitor renewal and lung fibrosis during aging and in IPF are incompletely understood. We have previously demonstrated that AEC2 progenitor function is impaired in IPF (13), and our goal was to further identify potential mechanisms contributing to this defect. In this study, we identified reduced expression of *SLC39A8*/ZIP8 in AEC2s in lungs of patients with IPF using single-cell transcriptome analysis. We further observed reduced expression of cell-surface ZIP8 and decreased intracellular zinc content in IPF AEC2s. Most notably, we showed that ZIP8 plays an important role in regulating AEC2 progenitor renewal. Zinc treatment promoted renewal of AEC2s from both healthy and IPF lungs in 3D organoid cultures. These are the first data to our knowledge to show that zinc metabolic homeostasis is crucial for maintaining proper progenitor function of AEC2s, and we have identified a defect of zinc metabolism within IPF AEC2s, as depicted in a summary schematic (Figure 7).

Our findings with AEC2s from human diseased lungs were corroborated well by the results from animal model studies with scRNA-Seq, in vitro culture, and in vivo lung fibrosis. With the animal model of aging, we found that the ZIP8 expression and renewal capacity of AEC2s from lungs of old mice were reduced relative to those of AEC2s from young mice. Zinc treatment promoted renewal of AEC2s from young mice, while the response to zinc diminished with AEC2s from older mice. These data demonstrate that decreased ZIP8 expression was associated with aging, and that contributed to the impaired renewal capacity of AEC2s. We generated a mouse line and showed that targeted deletion of ZIP8 in the AEC2 compartment led to reduced AEC2 renewal, spontaneous lung fibrosis with age, and increased lung fibrosis after injury, further confirming the role of ZIP8 in regulating AEC2 progenitor function and lung fibrosis.

There are multiple factors that might contribute to the impaired progenitor function of AEC2s during aging, including apoptosis (19), endoplasmic reticulum (ER) stress (20), senescence (22), and mitochondrial dysfunction (25). Our study adds ZIP8 deficiency as another contributor to the debilitated progenitor function of AEC2s in the aged lung. Zinc deficiency in the elderly has been reported (30). Zinc metabolism may influence alveolar progenitor activities by a variety of downstream mechanisms. One mechanism suggested by our data from pathway analysis in IPF AEC2s is zinc-dependent sirtuin signaling. We observed downregulation of sirtuin signaling in IPF AEC2s relative to AEC2s from healthy lungs. Using 3D Matrigel organoid cultures, we showed that sirtuin activation enhanced, whereas sirtuin inhibition suppressed. colony formation of AEC2s. These data indicate that sirtuin signaling is crucial for optimal AEC2 progenitor renewal through a zinc-dependent mechanism. We further showed that ZIP8^–^ AEC2s had lower SIRT1 expression than ZIP8^+^ cells and that zinc treatment augmented SIRT1 expression as well as the renewal capacity of AEC2s. We conclude that ZIP8 and zinc metabolism regulate AEC2 progenitor function through sirtuin signaling. Furthermore, the combination of zinc supplementation and SIRT1 activation had an additive effect on promoting renewal of AEC2s from IPF lungs. Interestingly, we also found that enzymes regulating NAD^+^ synthesis were also downregulated in IPF AEC2s. Exogenous supplementation with NAD^+^ precursors augmented AEC2 renewal in organoid assays. The optimal combinations of zinc, NAD^+^, and SIRT1 activation that may restore AEC2 renewal and mitigate fibrosis will require further studies.

It is intriguing to contemplate the relationship among aging, zinc metabolism, alveolar progenitor renewal, and pulmonary fibrosis. The cumulative impact of genetic and epigenetic reprogramming during aging may lead to zinc metabolic dysregulation. Many transcription factors (58, 59) and epigenetic mediators such as histone deacetylases (HDACs; ref. 60), are zinc dependent. For example, ZIP8 is regulated by NF-κB (58), whereas zinc regulates NF-κB activity (61). Both HDACs and histone H1 polypeptides are substrates of sirtuins (60, 62). We have previously shown that NF-κB activation in AEC2s impacts repair of lung injury (63). In addition, we identified a role for TLR4 and hyaluronan synthase 2 (HAS2) in regulating AEC2 renewal and identified a loss of cell-surface hyaluronan on IPF AEC2s (13). Interestingly, exogenous zinc supplementation increased cell-surface hyaluronan expression, suggesting that HAS2 may also be a downstream target of zinc metabolism (data not shown). Thus, a zinc-deficient state could fuel the dysregulation of genetic and epigenetic reprogramming. These compounding factors would further stress alveolar progenitors, leading to loss of alveolar epithelial integrity and proper repair after injury.

In summary, we have uncovered a mechanism by which the ZIP8/sirtuin axis regulates AEC2 renewal in vitro and pulmonary fibrosis in vivo. Furthermore, we have identified a selective loss of ZIP8 in AEC2s during aging and in IPF that results in disrupted sirtuin function, impaired AEC2 progenitor renewal, and lung fibrosis. Elucidating the proximal molecular regulation of the ZIP8/sirtuin axis and the role of zinc as a critical nutrient in lung repair could contribute to the identification of novel approaches to rejuvenating AEC2 renewal, reversing fibrosis, and improving lung function and survival for patients with IPF.

## Methods

### Study with cells from donor lung tissues.

The study included 21 healthy participants (12 male and 9 female) and 28 participants with IPF (22 male and 6 female). The median age of healthy donors was 60, and the median age of those with IPF was 66.

### Human lung dissociation and flow cytometry.

Human lung single-cell isolation and flow cytometry analysis were performed as described previously (10, 13). In brief, human lung tissues were minced and then digested with 2 mg/mL Dispase II, followed by digestion with 10 U/mL elastase and 100 U/mL DNase I. Finally, cells were filtered through a 100 μm cell strainer and lysed with red blood cell lysis to get single-cell suspensions. Antibody staining was similar in human and mouse cells. Flow cytometry was performed with a Fortessa and FACSAria III flow cytometer and analyzed with FlowJo 10.6.1 software (BD Biosciences). Anti–human CD31 (clone WM59, catalog 303118, RRID AB_2247932), CD45 (clone WI30, catalog 304016, RRID AB_314404), and EpCAM (clone 9C4, catalog 324212, RRID AB_756086) were from BioLegend. SLC39A8 (ZIP8) polyclonal antibody (catalog PA5-26368, RRID AB_2543868) and goat anti–mouse IgG/IgM (catalog A-10680, RRID AB_2534062) were from Thermo Fisher Scientific. HTII-280 (52) was a gift from the laboratory of L. Dobbs (UCSF). Human SIRT1 monoclonal mouse IgG1 (clone 834918, catalog IC7714S) from R&D Systems was used for intracellular staining. Pirfenidone was from MilliporeSigma (catalog P2116), and nintedanib was from Selleck Chemicals (catalog S1010).

### scRNA-Seq.

scRNA-Seq was performed at the Cedars-Sinai Medical Center Genomic Core. In brief, flow-sorted human lung single cells were lysed, and mRNA was reverse transcribed and amplified as previously described (37). Barcoding and library preparation were done with standard procedures according to manufacturer’s manuals (10x Genomics). The barcoded libraries were sequenced with NextSeq500 (Illumina) to obtain a sequencing depth of approximately 200,000 reads per cell.

Raw scRNA-Seq data were aligned to human genome GRCh38 and mouse genome mm10 with Cell Ranger (10x Genomics), respectively. Downstream quality control, normalization, and visualization were done with a Seurat package (37, 38). For quality control, the output expression matrix from Cell Ranger was done based on the number of genes detected in each cell, number of transcripts detected in each cell, and percentage of mitochondrial genes. The expression matrix was then normalized and visualized with UMAP.

scRNA-Seq on flow-sorted Lin^–^EpCAM^+^ cells from lungs of 2-month-old (young) and 18-month-old mice was reported recently (54). scRNA-Seq was performed on magnetically enriched CD45^–^ cells from 10- to 12-week-old Zip8^AEC2^ mice and littermate controls 2 weeks after 4 doses of tamoxifen injection. scRNA-Seq data sets from other groups, including GSE135893 (40), GSE132915 (38), GSE132771 (41), and GSE128033 (42), were used to determine SLC39A8 expression in IPF, in addition the data generated in-house.

Ingenuity Pathway Analysis (IPA) was performed as described previously (37). Genes differentially expressed between healthy and IPF AEC2s with a log fold change greater than 0.1 were analyzed with R software. The data were then imported into IPA software (QIAGEN). Canonical pathways from the core analysis were further analyzed. The activation score reflects the sum of expression levels of genes related to a biological process (or pathway) in each single cell; the results for each cluster of cells are shown in violin plots as previously described (38). The genes in the biological process (or pathway) were downloaded from UniProt (https://www.uniprot.org).

### Mice.

All mice were housed in a pathogen-free facility at Cedars-Sinai Medical Center. *SFTPC-CreER* mice and *Rosa-Tomato^fl/fl^* mice were described previously (10). *Slc39a8^fl/+^* mice were generated using the Cre/loxP system and acquired from the MMRRC Repository. All mice were backcrossed on a C57BL/6J background for more than 6 generations. Eight- to 12-week-old (young) and 18- to 20-month-old (old) WT C57BL/6J mice were obtained from the Jackson Laboratory and housed in the institution facility at least 2 weeks before experiments. Animals were randomly assigned to treatment groups, and they were age- and sex-matched.

The mouse diet was from LabDiet with customized zinc levels: the control diet contained 80 ppm zinc (which is the same level of zinc as the commonly used mouse chow LabDiet 5053); the zinc-deficient diet, 20 ppm; and the zinc supplement diet, 120 ppm.

### Bleomycin instillation.

Bleomycin instillation was described previously (13). Under anesthesia, the mouse trachea was surgically exposed. 1.25–2.5 U/kg bleomycin (Hospira) in 25 μL PBS was instilled into the trachea with a 25-G needle inserted between the cartilaginous rings of the trachea. Control animals received saline alone. The tracheostomy site was sutured, and the animals were allowed to recover. Mice were sacrificed at different time points, and lung tissue was collected for experiments.

### Hydroxyproline.

Collagen content in mouse lungs was measured with a conventional hydroxyproline method (64). In brief, lung tissues were vacuum dried and hydrolyzed with 6N hydrochloride acid at 120°C overnight. Hydroxyproline content was measured and expressed as mg per lung. The ability of the assay to completely hydrolyze and recover hydroxyproline from collagen was confirmed using samples containing known amounts of purified collagen.

### Lung tissue and histology.

Mice were sacrificed at various time points with or without bleomycin treatment under anesthesia. The trachea was cannulated, and the lung was inflated with 1.0 mL of 10% neutral buffered formalin. Mouse lung tissue was fixed, embedded in paraffin, and sectioned to 5 μm slices for H&E and Masson’s trichrome staining (64).

### Immunofluorescence staining of human lung sections.

Cryosections were used for the staining. AEC2s were stained with HTII-280 (52) (gift from the L. Dobbs laboratory) and goat anti–mouse IgG/IgM (Thermo Fisher Scientific, catalog A-10680, RRID AB_2534062). ZIP8 was stained with ZIP8 polyclonal antibody (Thermo Fisher Scientific, catalog 20459-1-AP) and Cy3 AffiniPure Donkey Anti-Rabbit IgG (Jackson ImmunoResearch Laboratories Inc., catalog 711-165-152, RRID AB_2307443).

### Mouse lung dissociation, flow cytometry, and magnetic sorting.

Mouse lung single-cell suspensions were isolated as previously described (13). In brief, lungs were perfused with 10 mL PBS, then digested with 4 U/mL elastase (Worthington Biochemical Corp.) and 100 U/mL DNase I (MilliporeSigma) and resuspended in HBSS supplemented with 2% FBS, 10 mM HEPES, 0.1 mM EDTA (HBSS+ buffer). The cell suspension was incubated with primary antibodies including CD24-PE; EpCAM–PE-Cy7; Sca-1–APC; biotinylated CD31, CD34, and CD45; as well as conjugated ZIP8 (rabbit IgG) for 45 minutes. Biotin-conjugated antibodies were detected following incubation with streptavidin-APC-Cy7 (catalog 405208, BioLegend), while ZIP8 was detected with goat anti–rabbit IgG–FITC for 30 minutes. Dead cells were discriminated by 7-amino-actinomycin D (7-AAD) (BD Biosciences) staining. Flow cytometry was performed using a Fortessa flow cytometer and FACSAria III sorter (BD Immunocytometry Systems) and analyzed using FlowJo 10.6.1 software.

Primary antibodies EpCAM–PE-Cy7 (clone G8.8, catalog 118216, RRID AB_1236471) and Ki-67 (clone 16A8, catalog 652403, AB_2561524) were from BioLegend. CD24-PE (clone M1/69, catalog 12-0242-82, RRID AB_467169), Sca-1 (Ly-6A/E)–APC (clone D7, catalog 17-5981-82, RRID AB_469487), CD31 (PECAM-1) (clone 390, catalog 13-0311-85, RRID AB_466421), CD34 (clone RAM34, catalog 13-0341-85, RRID AB_466425), and CD45 (clone 30-F11, catalog 13-0451-85, RRID AB_466447) were from eBioscience. SLC39A8 polyclonal antibody (catalog PA5-26368, RRID AB_2543868) was from Thermo Fisher Scientific.

Magnetic enrichment of CD45^–^ cells from Zip8^AEC2^ mice and control mice was performed with anti–mouse CD45 microbeads (MACS, catalog 130-052-301, Miltenyi Biotec) as previously described (65).

### Intracellular staining for zinc of human and mouse AEC2s.

The zinc assay kit (catalog ab241014) was from Abcam. Single cells isolated from human and mouse lungs were cultured on collagen IV–coated plates with 100 μM ZnSO_4_ overnight. Cells were stained for intracellular zinc following the manufacturer’s protocol, then AEC2 surface markers were stained. Flow cytometry was performed using a Fortessa flow cytometer. Intracellular zinc staining of gated human or mouse AEC2s was analyzed using FlowJo 10.6.1 software.

### 3D organoid cultures of human and mouse AEC2s.

Flow-sorted human (EpCAM^+^HTII-280^+^) or mouse (EpCAM^+^CD24^–^Sca-1^–^) AEC2s were cultured in a Matrigel/medium (1:1) mixture in the presence of MLg2908 lung fibroblasts. 100 μL Matrigel/medium mix containing 3 × 10^3^ AEC2s and 2 × 10^5^ MLg2908 cells (catalog CCL-206, ATCC) were plated into each 0.4-μm-pore-size insert of 24-well Transwell plates. 400 μL medium was added in the lower chambers. Cells were cultured with medium alone or with the treatments indicated. Medium was described previously (13). Matrigel (Growth Factor Reduced Basement Membrane Matrix, catalog 354230) was from Corning Life Sciences. For cell treatment, the following chemicals were used. 100 μM ZnSO_4_, 1 μM *N*,*N*,*N′*,*N′*-tetrakis(2-pyridylmethyl)ethylenediamine (TPEN), and 100 μM splitomicin were from MilliporeSigma. 1 μM SRT1720 was from Selleck Chemicals. The NAD^+^ precursor NMN (100 μM, catalog N3501) was from MilliporeSigma. The NAD precursor NR (100 μM, catalog 23132) was from Cayman Chemical. The same volume of DMSO was used as control. Medium was refreshed every other day. Cultures were maintained in a humidified 37°C and 5% CO_2_ incubator. Colonies were visualized with a Zeiss Axiovert 40 inverted fluorescence microscope. The colonies with a diameter of ≥50 μm from each insert were counted, and CFE was determined by the number of colonies in each culture as a percentage of input epithelial cells 12 days after plating.

### Organoid size measurement.

Organoids derived from flow-sorted Rosa-Tomato^+^ cells from *SFTPC-CreER^+^ Rosa-Tomato^fl/fl^* mice and *SFTPC-CreER^+^ Rosa-Tomato^fl/fl^* *Slc39a8^fl/fl^* (Zip8^AEC2^) mice were imaged on day 12 after plating using a Zeiss Axiovert 40 inverted fluorescence microscope. ZEN Pro 2012 software (Zeiss) was used for measuring surface area of the imaged organoids as previously described (13).

### Cell lines.

MLg2908 mouse lung fibroblasts (catalog CCL-206) and A549 (catalog CCL-185) human adenocarcinoma alveolar epithelial cells were from ATCC. Mycoplasma contamination was assessed with a MycoFluor Mycoplasma Detection Kit (catalog M7006, Thermo Fisher Scientific), and cells used for experiments were free of mycoplasma contamination.

### RNA analysis.

RNA was extracted from mouse AEC2s or human AEC2s using TRIzol Reagent. For real-time PCR analysis, 0.5 μg total RNA was used for reverse transcription with the High Capacity cDNA Reverse Transcription Kit (Applied Biosystems). One microliter cDNA was subjected to real-time PCR by using Power SYBR Green PCR Master Mix (Applied Biosystems) and the ABI 7500 Fast Real-Time PCR system (Applied Biosystems). The specific primers were designed on the basis of cDNA sequences deposited in the GenBank database: human *SLC39A8* (NM_001135146), forward 5′-CATCTGTCCAGCAGTCTTACAGC-3′ and reverse 5′-GACAGGAATCCATATCCCCAAACT-3′; mouse *Slc39a8* (NM_001135149), forward 5′-AGCGATCCTGTGTGAGGAGT-3′ and reverse 5′-CGGAGAGGAAGTTGAACAGC-3′; mouse *Slc39a7* (NM_008202.2), forward 5′-AGCAAACCGAGAACCGAGAG-3′ and reverse 5′-GTTGGGGTAAAGGCCTGGAA; human *SFTPC* (NM_003018.4), forward 5′-ATCCCCAGTCTTGAGGCTCT-3′ and reverse 5′-CTTCCACTGACCCTGCTCAC-3′; mouse *Sftpc* (NM_011359.2), forward 5′-GCAGGTCCCAGGAGCCAGTTC-3′ and reverse 5′-GGAGCTGGCTTATAGGCCGTCAG-3′; human *PDPN* (NM_006474.5), forward 5′-GGAAGGTGTCAGCTCTGCTC-3′ and reverse 5′-CGCCTTCCAAACCTGTAGTC-3′; mouse *Pdpn* (NM_010329.3), forward 5′-GCACCTCTGGTACCAACGCAGA-3′ and reverse 5′-TCTGAGGTTGCTGAGGTGGACAGT-3′; mouse Slc39a7 (NM_008202.2), forward 5′-AGCAAACCGAGAACCGAGAG-3′ and reverse 5′-GTTGGGGTAAAGGCCTGGAA-3′; human *SIRT1* (NM_012238.5), forward 5′-TGCCGGAAACAATACCTCCA-3′ and reverse 5′-AGACACCCCAGCTCCAGTTA-3′; mouse *Sirt1* (NM_019812.3), forward 5′-GAGCTGGGGTTTCTGTCTCC-3′ and reverse 5′-CTGCAACCTGCTCCAAGGTA-3′; human *GAPDH* (NM_002046), forward 5′-CCCATGTTCGTCATGGGTGT-3′ and reverse 5′-TGGTCATGAGTCCTTCCACGATA-3′; mouse *Gapdh* (NM_00100130), forward 5′-ATCATCTCCGCCCCTTCTG-3′ and reverse 5′-GGTCATGAGCCCTTCCACAAC-3′. The relative expression level of each gene was determined relative to the GAPDH level in the same sample. The fold change of the target genes was calculated by using the 2^–ΔΔCT^ method.

*SLC39A8* expression was also compared in IPF and healthy control participants using the LGRC data set GEO GSE47460 (44). Furthermore, the correlation between *SLC39A8* expression and DLCO was determined with samples from participants with available lung function data.

### Generation of the SIRT1-KO cell line with CRISPR/Cas9.

Generation of SIRT1-KO A549 cells with the CRISPR/Cas9 system was as previously described (65). Two sets of sgRNA sequences were designed to knock out SIRT1 in A549 cells. Set 1 sgRNA sequences were 5′-TACCCAGAACATAGACACG-3′ and 5′-GCTGGGCACCTAGGACATCG-3′. Set 2 sgRNA sequences were 5′-GTTGACTGTGAAGCTGTACG-3′ and 5′-TTACTTGGAATTAGTGCTA-3′. Nested PCR with the following primers was conducted to validate SIRT1 knockout at the genome level: primer set 1, forward 5′-CTTCAAGGGGCCAAGTTCA-3′ and reverse 5′-CCACTCTTTCAAACAGAAGCAGAG-3′; primer set 2, forward 5′-TCCTGGACAATTCCAGCCAT-3′ and reverse 5′-CCACTCTTTCAAACAGAAGCAGAG-3′.

### Western blot analysis.

Proteins were assessed with Western blotting as previously described (64). The membranes were probed with antibodies against SIRT1 (catalog PA5-85921, Thermo Fisher Scientific). β-Actin (catalog 12620, Cell Signaling Technology) was used as a loading control.

### Material transfer agreement.

All unique/stable reagents generated in this study are available from the corresponding authors with a completed material transfer agreement (MTA).

### Data and code availability.

The raw data files of the scRNA-Seq were deposited in the NCBI’s Gene Expression Omnibus database as GSE157997 and GSE187027. R code files used for data integration and analysis are available at https://github.com/jiang-fibrosis-lab/Zip8-function-in-AEC2 (commit IDs 8c3ebab, b11d5a6, and 07a8e3b). Other scRNA-Seq data used in this study can be accessed via data sets GSE135893 (40), GSE132915 (38), GSE132771 (41), and GSE128033 (42). The LGRC data set was GSE47460 (44).

### Statistics.

The statistical difference between groups in the bioinformatics analysis was calculated using Wilcoxon’s signed-rank test. For the scRNA-Seq data, the lowest *P* value calculated in Seurat was *P* < 2.2 × 10^–16^. For all other data, the statistical difference between groups was calculated using Prism (version 8.4.3, GraphPad Software) and the exact value shown. Data are expressed as mean ± SEM. The sample size for in vivo bleomycin fibrosis studies was based on previous studies in our laboratory (13, 64). No animals were excluded for analysis. All experiments were repeated 2 or more times. Data were normally distributed, and the variance between groups was not significantly different. Differences in measured variables between experimental and control groups were assessed by using Student’s *t* tests. One-way ANOVA followed by Bonferroni’s or Dunnett’s multiple-comparison test or 2-way ANOVA followed Tukey’s multiple-comparison test was used for multiple comparisons. Results were considered statistically significant at *P* < 0.05.

### Study approval.

Use of human tissues for research was approved by the Cedars-Sinai Medical Center Institutional Review Board (Pro00032727) and UCLA Institutional Review Board (13-000462-AM-00019). Informed consent was obtained from each participant. All mouse experiments were approved by the Institutional Animal Care and Use Committee of Cedars-Sinai Medical Center (IACUC008529).

## Author contributions

JL, DJ, and PWN conceived the study. JL performed most of the experiments and analyzed the data. GH analyzed single-cell RNA transcriptome data, performed flow cytometry analysis, designed and performed CRISPR/Cas9 knockout experiments, and prepared figures. JL, GH, XL, YW, ND, CY, and DJ analyzed single-cell RNA transcriptome data. XL, FT, NL, TX, VK, KD, AB, and SCR took part in mouse, cell culture, and biological experiments. SSW and JB provided human samples and interpreted data. BS and WCP interpreted data and commented on the manuscript. JL, DJ, and PWN wrote the manuscript. All authors read and reviewed the manuscript.

## Supplementary Material

Supplemental data

## Version 1 04/07/2022

In-Press Preview

## Version 2 06/01/2022

Electronic publication
