# Supplementary material for: The ZIP8/SIRT1 axis regulates alveolar progenitor cell renewal in aging and idiopathic pulmonary fibrosis
Source: J Clin Invest. 2022 Jun 1;132(11):e157338. doi: 10.1172/JCI157338 (PMC9151700; doi:10.1172/JCI157338)

The ZIP8-SIRT1 axis regulates alveolar progenitor cell renewal in aging and idiopathic pulmonary fibrosis

Jiurong Liang,[1,*] Guanling Huang,[1] Xue Liu,[1] Forough Taghavifar,[1] Ningshan Liu,[1] Yizhou Wang,[2] Nan Deng,[2] Changfu Yao,[1] Ting Xie,[1] Vrishika Kulur,[1] Kristy Dai,[1] Ankita Burman,[1] Simon Rowan,[1] S. Samuel Weigt,[3] John Belperio,[3] Barry Stripp,[1] William C. Parks,[1,4] Dianhua Jiang,[1,4] and Paul W. Noble[1,*]

Supplemental Figures 1 – 7

Supplemental Figure 1. Specific expression of *SLC39A8* in human alveolar epithelial cells
Supplemental Figure 2. ZIP8 expression in human AEC2s in healthy and IPF lungs.
Supplemental Figure 3. Flow cytometry gating strategy for AEC2 intracellular zinc
Supplemental Figure 4. Flow sorting and 3D organoid culture of A549 cells with SIRT1 knockout
Supplemental Figure 5. Decreased ZIP8 expression and renewal capacity of old mouse AEC2s
Supplemental Figure 6. Down regulated gene expression of NAD synthesis enzymes in IPF AEC2s and NAD+ precursors promoted AEC2 renewal
Supplemental Figure 7. No obvious morphological defect in Zip8 deletion mice

Full unedited gels for Figure 3M

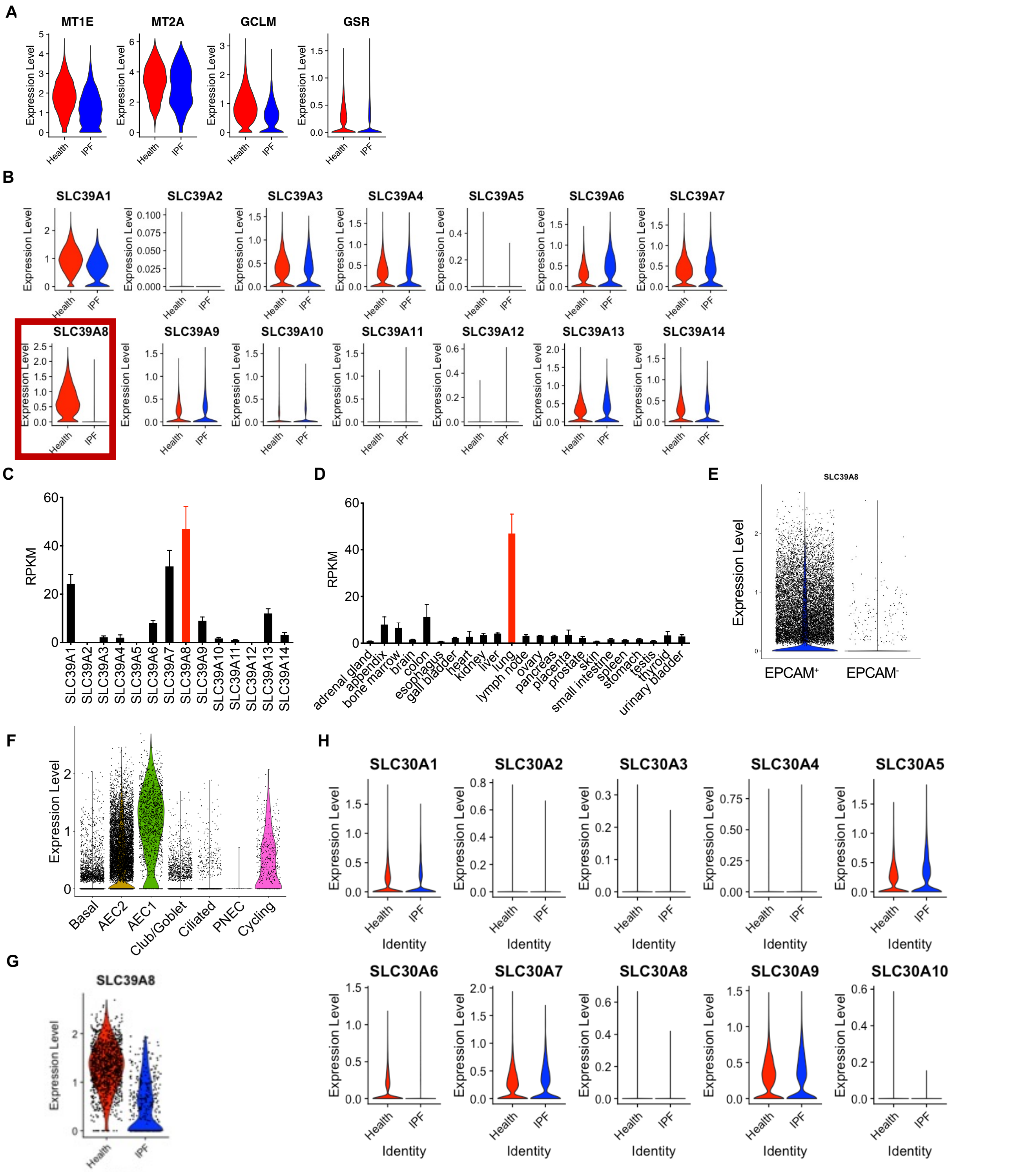

Supplemental Figure 1. Specific expression of *SLC39A8* in human alveolar epithelial cells. (**A**) Expression of zinc metabolism related genes in healthy and IPF AEC2s. (**B**) Expression of zinc transporter genes *SLC39A1-14* in AEC2s from healthy and IPF lungs, *SLC39A8* was boxed. (**C**) Expression of *SLC39A1-14* in lung tissue in dataset (PRJEB4337) deposited by Fagerberg et al. ref [43]. (**D**) Expression of *SLC39A8* in human tissues and organs in dataset (PRJEB4337). (**E**) *SLC39A8* expression in CD45$^-$EpCAM$^+$ and CD45$^-$EpCAM$^-$ cells in human lungs. (**F**) *SLC39A8* expression epithelial cell types in human lung. (**G**) Expression of *SLC39A8* in AEC1s from healthy and IPF lungs. (**H**) Expression of ZNT family genes *SLC30A1-10* in AEC2s from healthy and IPF lungs.

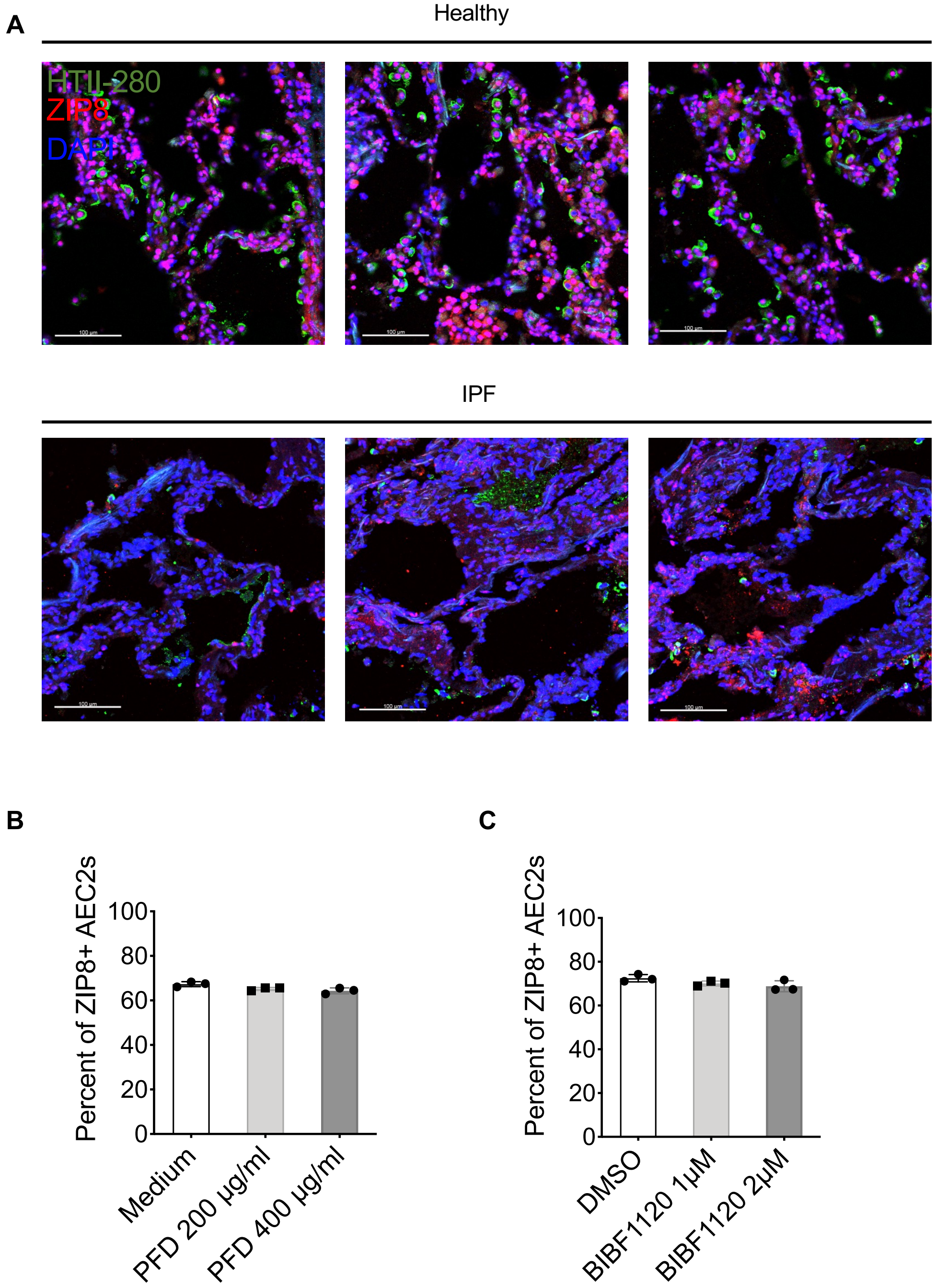

Supplemental Figure 2. ZIP8 expression in human AEC2s in healthy and IPF lungs. (**A**) Immunofluorescence staining of lung sections from healthy donors and IPF patients. Green, HTII-280 and red, ZIP8. Scale bars, 100 μM. (**B**,**C**) Flow cytometry, percent of ZIP8$^+$ cells in total gated AEC2s from healthy human lungs treated with pirfenidone (PFD) (**B**) and nintedanib (BIBF1120) (**C**) for 48 hours at indicated concentrations (n = 3).

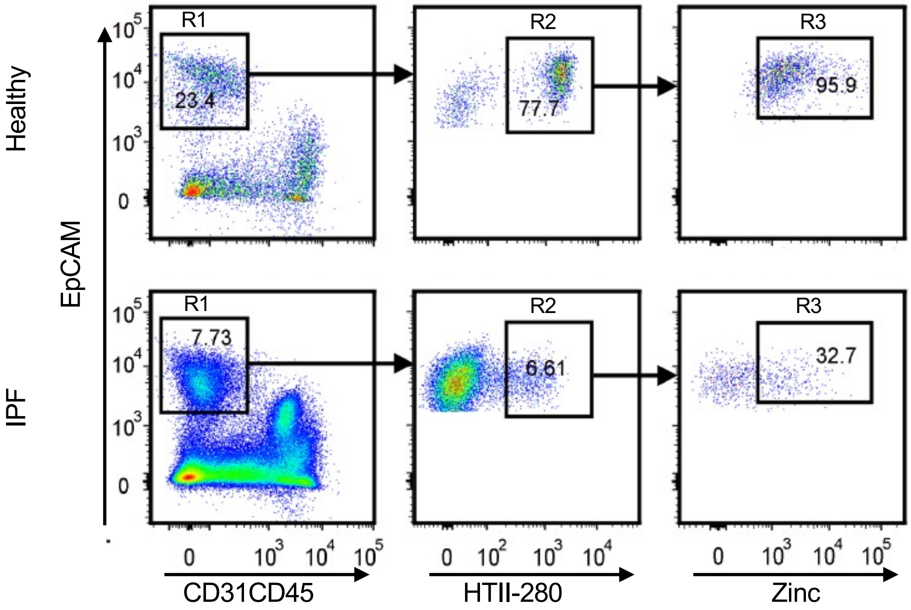

Supplemental Figure 3. Flow cytometry gating strategy for AEC2 intracellular zinc. Single cell homogenates from dissociated healthy and IPF lungs were cultured with 100 μM ZnSO4 for overnight and then stained with cell surface epithelial markers and intracellular zinc for flow cytometry. Total lung epithelial cells were gated in R1. Human AEC2s were defined as EPCAM+CD31-CD45-HTII-280+ in R2. Zinc+ AEC2s were gated in R3.

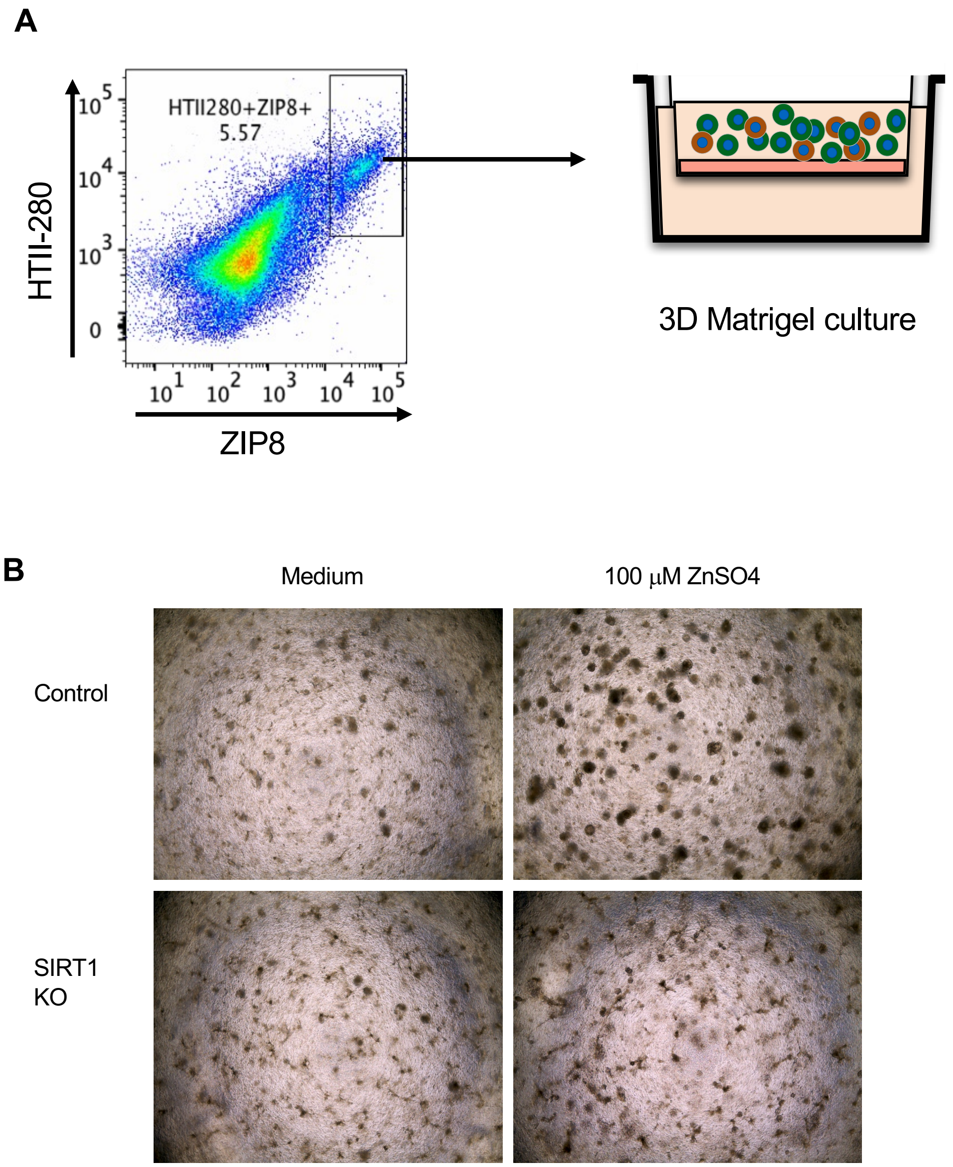

Supplemental Figure 4. Flow sorting and 3D organoid culture of A549 cells with SIRT1 knockout. (**A**) HTII-280 and ZIP8 double positive cells were sorted from SIRT1 knockout and control A549 cells for 3D organoid culture. (**B**) Representative pictures of colonies of SIRT1 knockout and control A549 cells (n = 4).

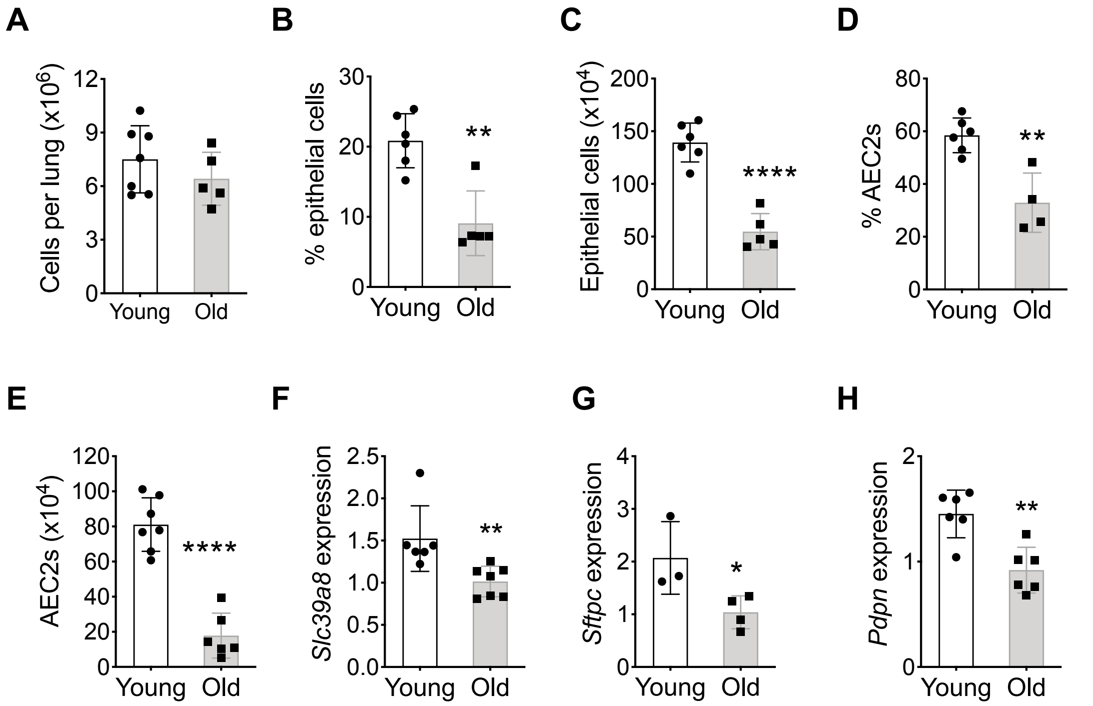

Supplemental Figure 5. Decreased ZIP8 expression and renewal capacity of old mouse AEC2s. (**A**). Number of total cells recovered from each lung of young and old mice (n = 5 – 6). (**B**,**C**) Percent (**B**) and number (**C**) of EpCAM$^+$CD31$^-$CD34$^-$CD45$^-$ epithelial cells (R1 in Figure 4**A**) (n = 5-6. **p< 0.01, ****p < 0.0001) in cells dissociated from young and old mouse lungs. (**D**) Percent of CD24$^-$Sca-1$^-$ AEC2s (R2 in Fig. 4a) within total lung epithelial cells (n = 4 – 6, **p < 0.01). (**E**) Number of AEC2s were recovered from each lung of young and old mice (n = 6 – 7, ****p < 0.0001). (**F**) *Slc39a8* expression in AEC2s from uninjured mouse lungs by qPCR (n = 6 – 7, **p< 0.01). (**G**, **H**) Expression of *Sftpc* (**G**) (n = 3 – 4, *p < 0.05) and *Pdpn* (**H**) (n = 6 each, **p< 0.01) of mouse AEC2s derived from 3D cultured organoids by RT-PCR. Data are shown as the mean ± SEM.

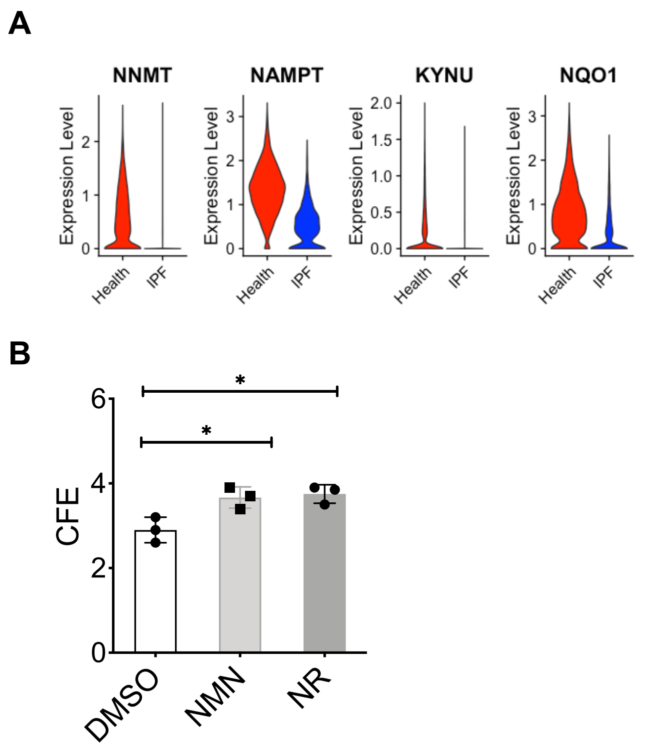

Supplemental Figure 6 . Down regulated gene expression of NAD synthesis enzymes in IPF AEC2s and NAD+ precursors promoted AEC2 renewal. (**A**) Violin plots of gene expression in AEC2s from healthy and IPF lungs. (**B**) CFE at 14 dpp of AEC2s from young wild type mice with NMN (100 μM), NR (100 μM) treatment and DMSO control (n = 3, *p< 0.05 by ANOVA). Data are shown as the mean ± SEM.

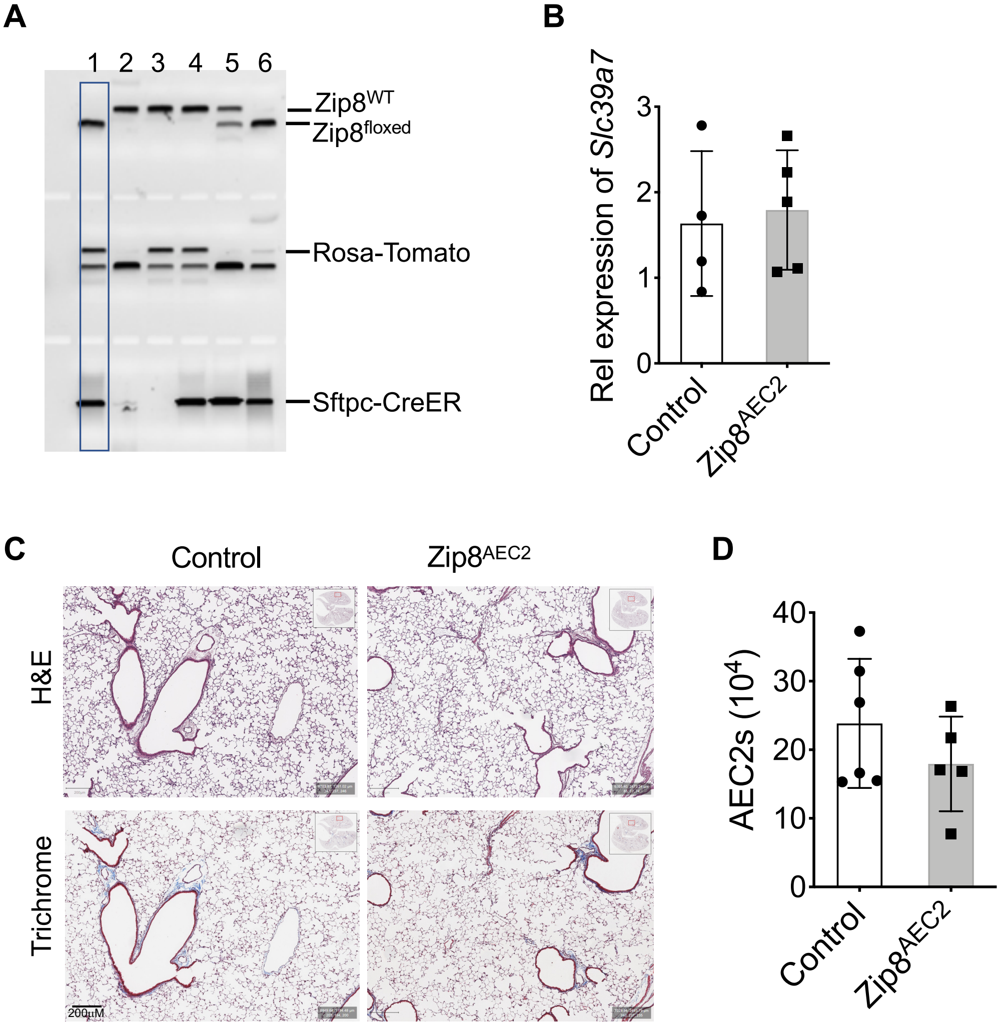

Supplemental Figure 7. No obvious morphological defect in Zip8 deletion mice. (**A**) representative genotyping result of *Sftpc-CreER*$^{+}$*;RosaTomato*$^{+}$*;Zip8*$^{flox/flox}$ (Zip8^{AEC2}) mice. (**B**) qPCR Slc39a7 expression in AEC2s from Zip8^{AEC2} and control mice (n = 4 - 5, p = 0.7671). (**C**) Histological characterization with H&E and trichrome staining of lung sections from 16 weeks old Zip8^{AEC2} and control mice after four doses of tamoxifen. Scale bars, 200 μM. (**D**) Number of AEC2s recovered from four dose tamoxifen injected young Zip8^{AEC2} (n = 5) and control mice (n = 6) without bleomycin injury (p = 0.275). Data are shown as the mean ± SEM.

Full unedited gels for Figure 3M

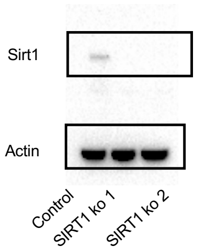

Original gels

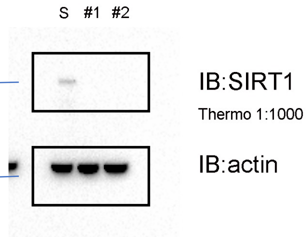

Original gels overlapped with background to show gel markers

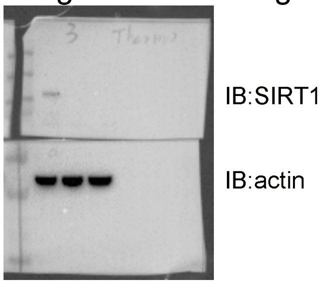

Supplement: Supplemental data [file jci-132-157338-s105.pdf]
